# Identification of key genes with differential correlations in prostate cancer

**DOI:** 10.18632/aging.206323

**Published:** 2025-10-10

**Authors:** Zepai Chi, Yuanfeng Zhang, Xuwei Hong, Tenghao Yang, Qingchun Xu, Weiqiang Lin, Yueying Huang, Yonghai Zhang

**Affiliations:** 1 Department of Urology, Shantou Central Hospital, Shantou 515031, Guangdong, P.R. China

**Keywords:** prostate cancer, DiffCorr, TCGA, biomarkers, WGCNA

## Abstract

Background: Prostate cancer, a major global health issue for men, remains a critical clinical challenge in treatment, highlighting the need for improved biomarkers. Treatment options for prostate cancer include active surveillance, surgery, endocrine therapy, chemotherapy, radiotherapy, immunotherapy, etc. However, as the tumor progresses, the effectiveness of treatment regimens gradually decreases. Therefore, we need to understand the biological mechanisms that promote prostate cancer tumorigenesis and progression and to screen biomarkers for diagnosis and prediction of prognosis.

Methods: We utilized the expression profiles of prostate cancer from The Cancer Genome Atlas (TCGA) database and employed weighted gene co-expression network analysis (WGCNA) to construct a gene interaction network. Gene co-expression networks were constructed using WGCNA (soft-threshold power β = 10, scale-free R² > 0.9), with differential correlations computed via Fisher’s *z*-test (FDR < 0.05). We used the “DiffCorr” package to discriminate between tumor and adjacent normal tissues to identify genes with differential representation in tumor and normal tissues, and perform in-depth analysis of these genes.

Results: Through WGCNA analysis, we identified a total of 20 modules, three gene modules were significantly associated with prostate cancer. We then analyzed the genes in these modules separately by the “DiffCorr” package and intersected these with differentially expressed genes. Finally, 21 genes were screened as biomarkers for prostate cancer.

Conclusions: Our study unveils a prostate cancer tumorigenesis mechanism by identifying differentially correlated gene pairs during normal-to-tumor transformation. We believe that the biomarkers derived from this algorithm have important reference implications for future research in prostate cancer.

## INTRODUCTION

Prostate cancer is one of the most common types of cancer in elderly men [1]. In recent years, the promotion of prostate cancer-based screening has increased the incidence of prostate cancer, while early detection has also reduced prostate cancer specific mortality [2]. In recent years, treatments based on androgen deprivation therapy (ADT) and radiotherapy have greatly improved the prognosis of patients [2, 3]. However, there is still a subset of patients with a poor prognosis. New approaches have been explored to improve patient outcomes, including androgen receptor signaling inhibitors (ARSI) [4] and immunotherapy [5, 6]. However, the prognosis of some patients with prostate cancer remains suboptimal. Therefore, it is also necessary to reveal the pathogenesis of prostate cancer more deeply with new biomarkers.

Currently, bioinformatics methods based on gene expression profiling have been developed to provide effective tools for comprehensive analysis of gene networks in cancer pathogenesis [7]. In recent years, much works studied and reported novel biomarkers with significant status in gene networks of different cancers, including hepatocellular carcinoma [8], breast cancer [9], non-small cell lung cancer [10], bladder cancer [11]. Recent work in bladder cancer [11] demonstrates the value of differential correlation approaches for uncovering network rewiring. In general, biomarkers are determined based on the analysis of differentially expressed genes between disease and healthy tissues. However, there is concern that the occurrence of a disorder is the combined effect of multiple highly interacting genes.

Correlation analysis is an important method for omics data to provide clues to gene regulatory networks [12]. Complementing traditional approaches to the analysis of gene expression data, it is critical to investigate how gene correlations (termed “differential correlations”) vary in cancer pathogenesis [13].

A recent paper reported a set of five dysregulated hub genes (MAF, STAT6, SOX2, FOXO1, and WNT3A) that play crucial roles in biological pathways associated with prostate cancer progression [14]. We constructed a gene correlation network for prostate cancer pathogenesis based on differential correlation theory for the first time and found some novel key genes. First, 20 modules of co-expressed genes were detected by weighted gene co-expression network analysis (WGCNA), in which 3 modules were significantly correlated with prostate cancer; Then, differentially correlated gene pairs in each module with the largest correlation to prostate cancer were calculated, gene networks were constructed and key genes were subjected to functional analysis. Finally, 21 biomarkers derived from web-based algorithm were screened, in which 5 genes have never been studied in prostate cancer research, including CPA6, KRT15, SMIM10, SPON1, and ST6GALNAC4.

## MATERIALS AND METHODS

### Data collection

The TCGA data of prostate cancer was downloaded from UCSC Xena database (https://xenabrowser.net/datapages/), including 499 tumor samples and 52 normal samples. The gene expression levels were quantified as FPKM (Fragments Per Kilobase of transcript per Million mapped reads) for subsequent analyses.

### WGCNA

The co-expression relationship of protein-coding genes was investigated by R package “WGCNA” to screen gene modules associated with prostate cancer. Gene modules most significantly associated (*p* < 0.05, r > 0.3) to prostate cancer were selected for subsequent analysis. R package “clusterProfiler” was used to conducted enrichment analysis of genes in each module.

### Differential correlation analysis

R package “DiffCorr” was utilized to identify and visualize differential correlations. This package was based on Fisher’s *z*-test and details were explained in published work [15].

### Statistical analysis

All data are presented as the mean ± SD (Standard Deviation). Statistical analysis was performed using R software (https://www.r-project.org/, version:4.1.1). *P* < 0.05 (two-tailed) was considered statistically significant: ^*^p < 0.05, ^**^p < 0.01, ^***^p < 0.001, and ^****^p < 0.0001.

### Data availability

The datasets generated and analysed during the current study are available from the corresponding author on reasonable request.

## RESULTS

### Identification of gene modules associated with prostate cancer

**Figure 1:**
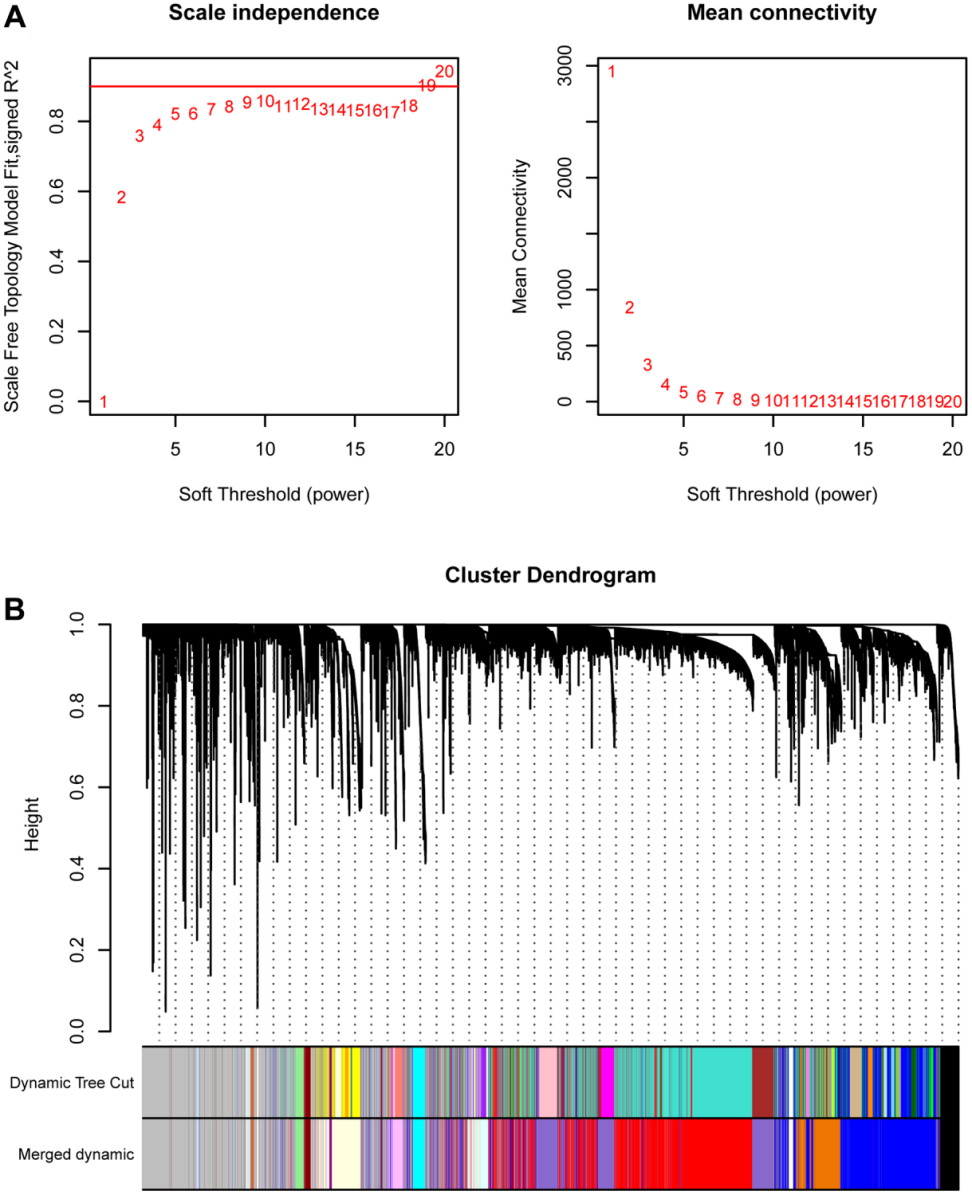
**WGCNA.** (**A**) The best soft threshold selection of WGCNA. (**B**) The combination of similar gene modules.

**Figure 2:**
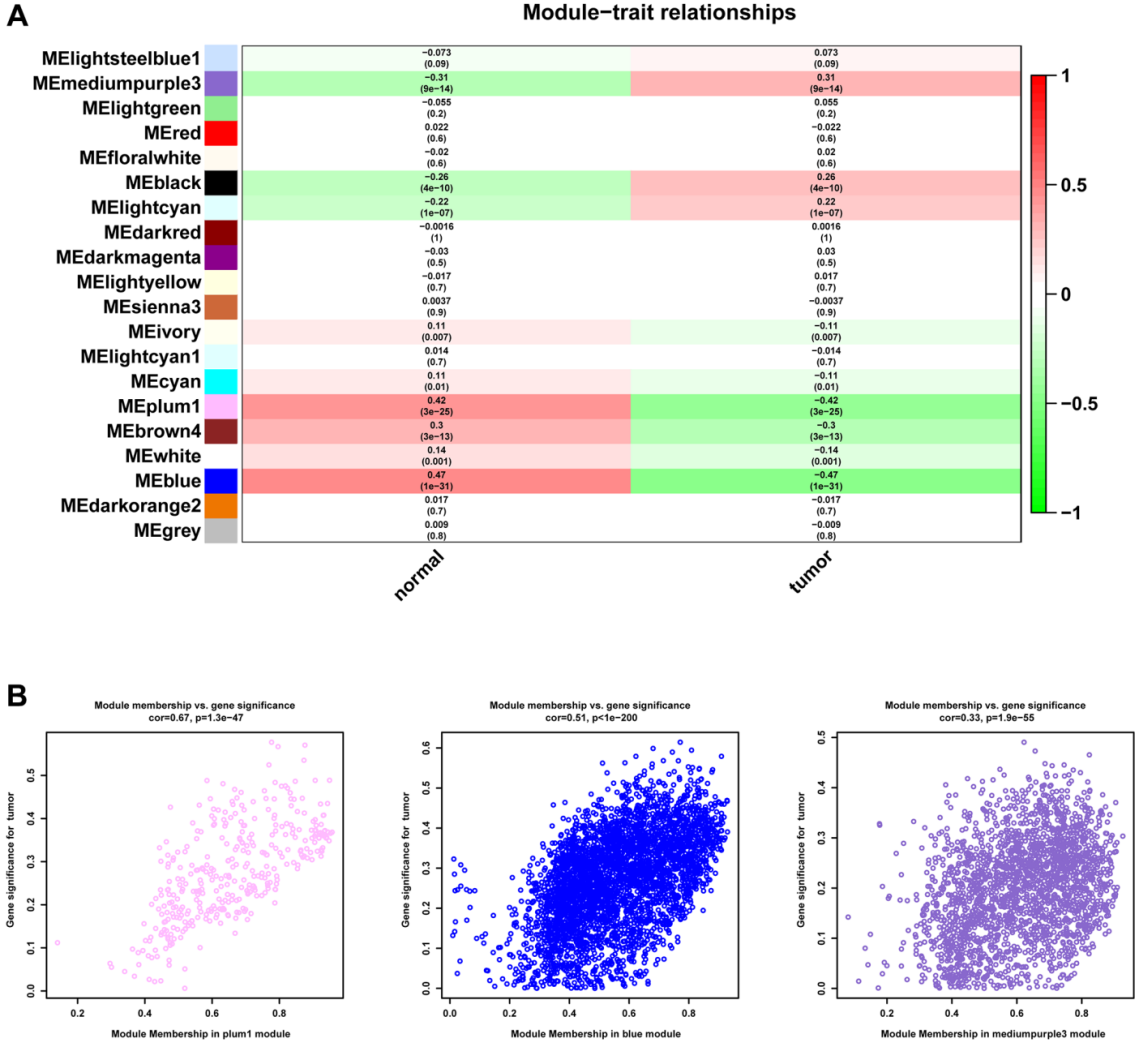
**Identification of gene modules.** (**A**) Heatmap of the correlation between module and the clinical features of patients in TCGA-PRAD. (**B**) The correlation between module membership and gene significance.

We first conducted WGCNA in TCGA-PRAD to screen key gene modules associated with prostate cancer. We performed weighted gene co-expression network analysis (WGCNA) using the following parameters: A soft-thresholding power (β) of 10 was selected based on scale-free topology fit (R² = 0.92) and mean connectivity preservation. This threshold optimally balances network connectivity with scale-free topology requirements. Minimum module size was set to 30 genes, Module merging threshold was 0.25 (Figure 1A). Figure 1B showed the merging of similar modules. MEplum1, MEblue, and MEmediumpurple3 gene modules (r > 0.3, *p* < 0.05) were selected for subsequent analysis (Figure 2A). Figure 2B displayed the correlation between module membership and gene significance.

**Figure 3:**
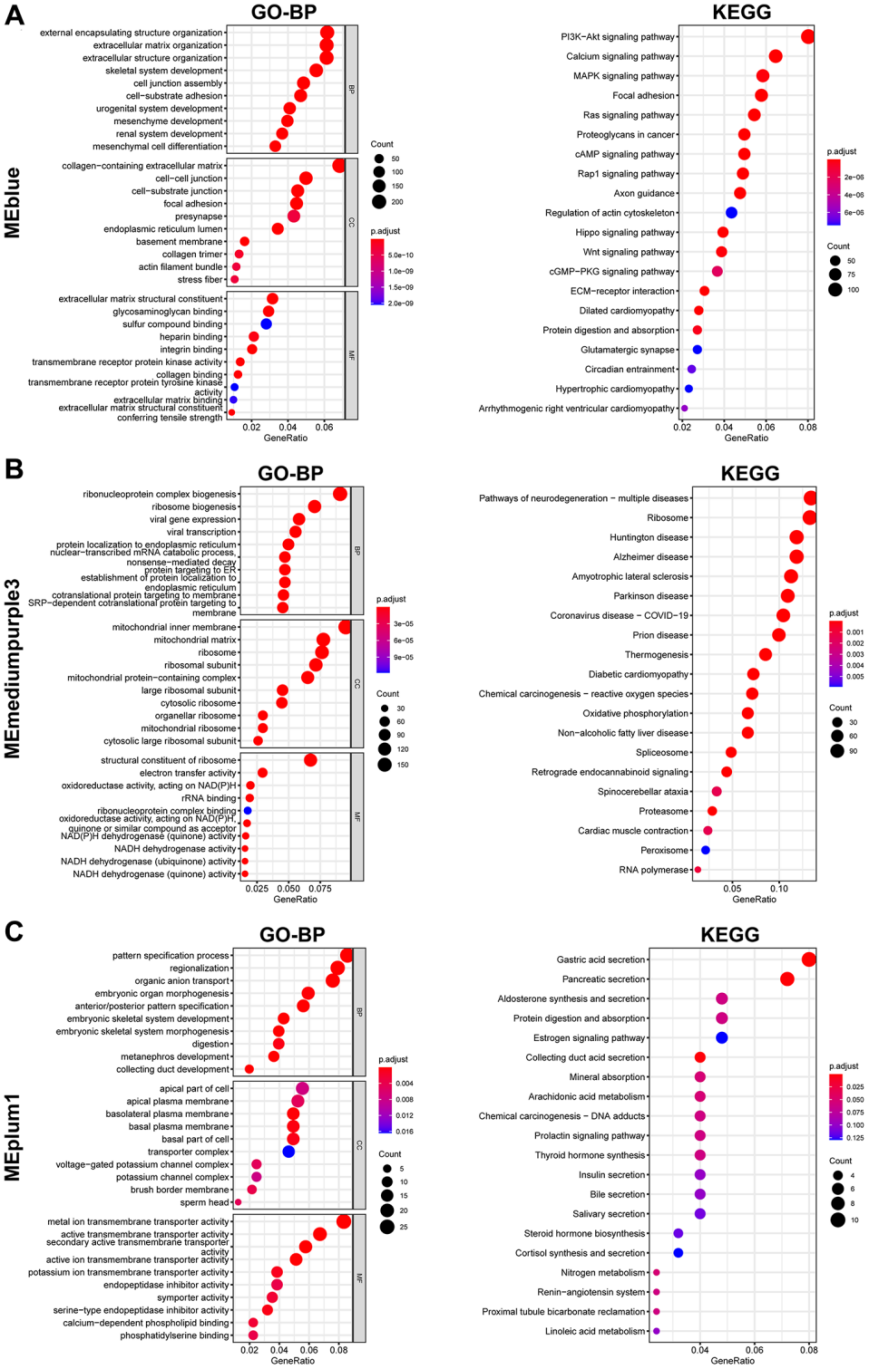
**Enrichment analysis of genes from selected modules.** (**A**–**C**) GO (Left) and KEGG (Right) enrichment analysis of genes in MEblue (**A**), MEmediumpurple3 (**B**), and MEplum1 (**C**).

GO and KEGG analyzes were further performed to explore the function of each gene module. Results indicated that these genes in MEblue were enriched in external encapsulating structure organization and extracellular matrix organization in BP (Biological Process) terms, collagen-containing extracellular matrix and cell-cell junction in CC (Cellular Component) terms, extracellular matrix structural constituent and glycosaminoglycan binding in MF (Molecular Function) terms, and PI3K-Akt signaling pathway in KEGG terms (Figure 3A). The MEblue module’s enrichment for PI3K-Akt signaling (Figure 3A) aligns with known pathway activation in prostate cancer metastasis. Genes in MEmediumpurple3 were enriched in ribonucleoprotein complex biogenesis in BP terms, mitochondrial inner membrane in CC terms, structural constituent of ribosome in MF terms, and Pathways of neurodegeneration-multiple diseases, Chemical carcinogenesis-reactive oxygen species, and Oxidative phosphorylation in KEGG terms (Figure 3B). Genes in MEplum1 were enriched in pattern specification process in BP terms, apical part of cell in CC terms, metal ion transmembrane transporter activity in MF terms, and Gastric acid secretion, Pancreatic secretion, and Aldosterone synthesis and secretion in KEGG terms (Figure 3C).

### Differential correlations identification

The genes in the MEplum1, MEblue, and MEmediumpurple3 modules were further selected to assess differential correlations via R package “DiffCorr”. Cluster.molecule algorithm was used to divide genes based on tumor and normal groups. We used the one-correlation coefficient as a distance measure (the cutoff was 0.5) according to the cutree function. Network analysis was performed using the DiffCorr package with the following key functions:

get.eigen.molecule: Computes module eigengenes (first principal components) representing each gene module’s expression pattern across samples. This dimensionality reduction approach captures >50% of variance in each module (mean = 62 ± 8%).

get.eigen.molecule.graph: Visualizes module relationships through force-directed layouts, where:

Nodes represent modules (size proportional to gene count).

Edges show significant inter-module correlations (|r| > 0.5, FDR < 0.05).

Colors indicate association strength with clinical traits.

**Figure 4:**
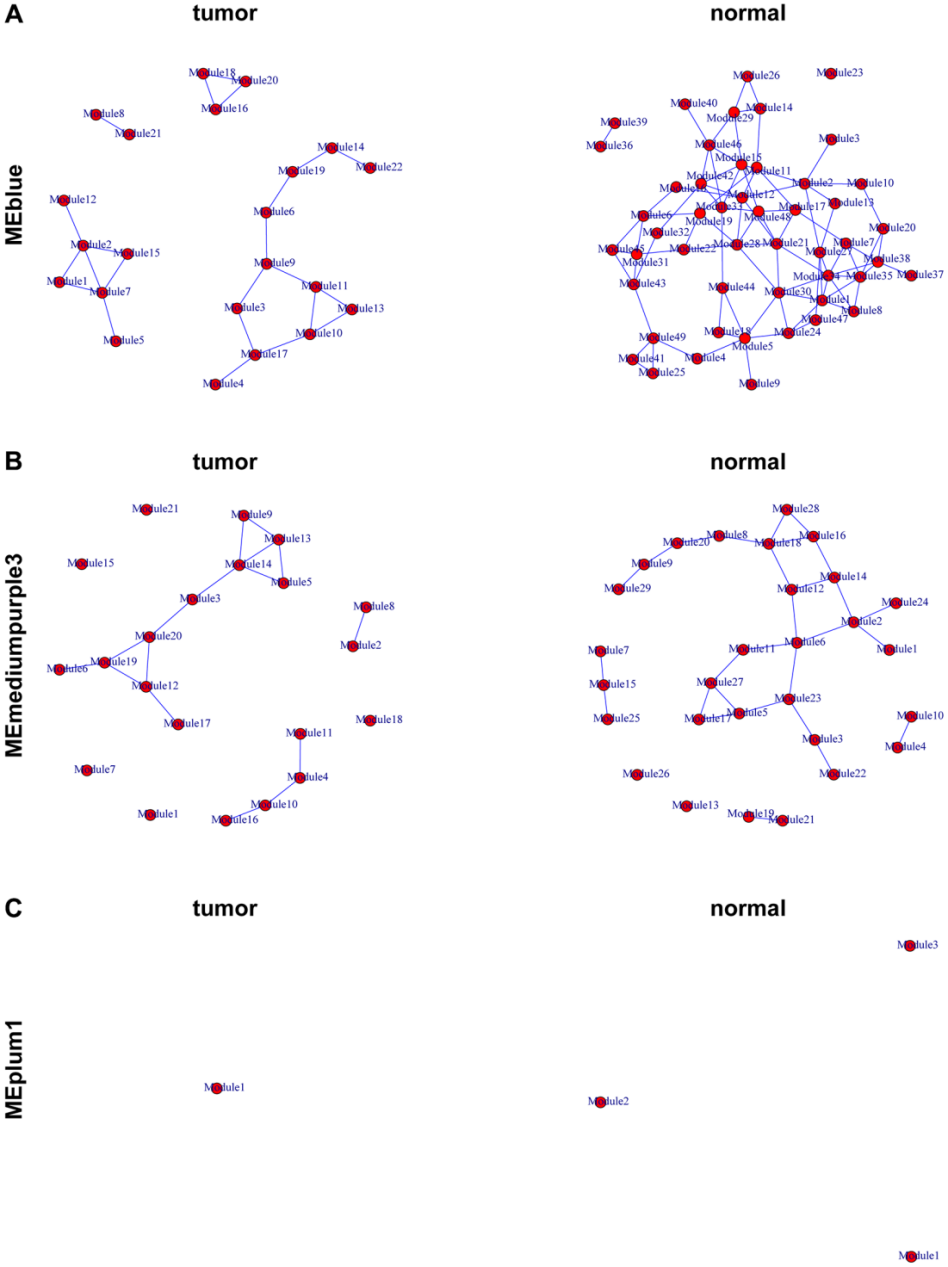
**Representation of the module networks.** Images of MEblue (**A**), MEmediumpurple3 (**B**), and MEplum1 (**C**) module networks from the TCGA-PRAD were shown. Each node represented one module, and each edge represented the module correlation.

The get.eigen.molecule and get.eigen.molecule.graph functions were used for visualization of the module network (Figure 4A–4C). The comp.2.cc.fdr function provided the resulting pair-wise differential correlations in each gene module. A total of 297 gene pairs were screened in MEblue module and 23 gene pairs were screened in MEmediumpurple3. No gene pairs were selected in MEplum1 (Supplementary Table 1).

**Figure 5:**
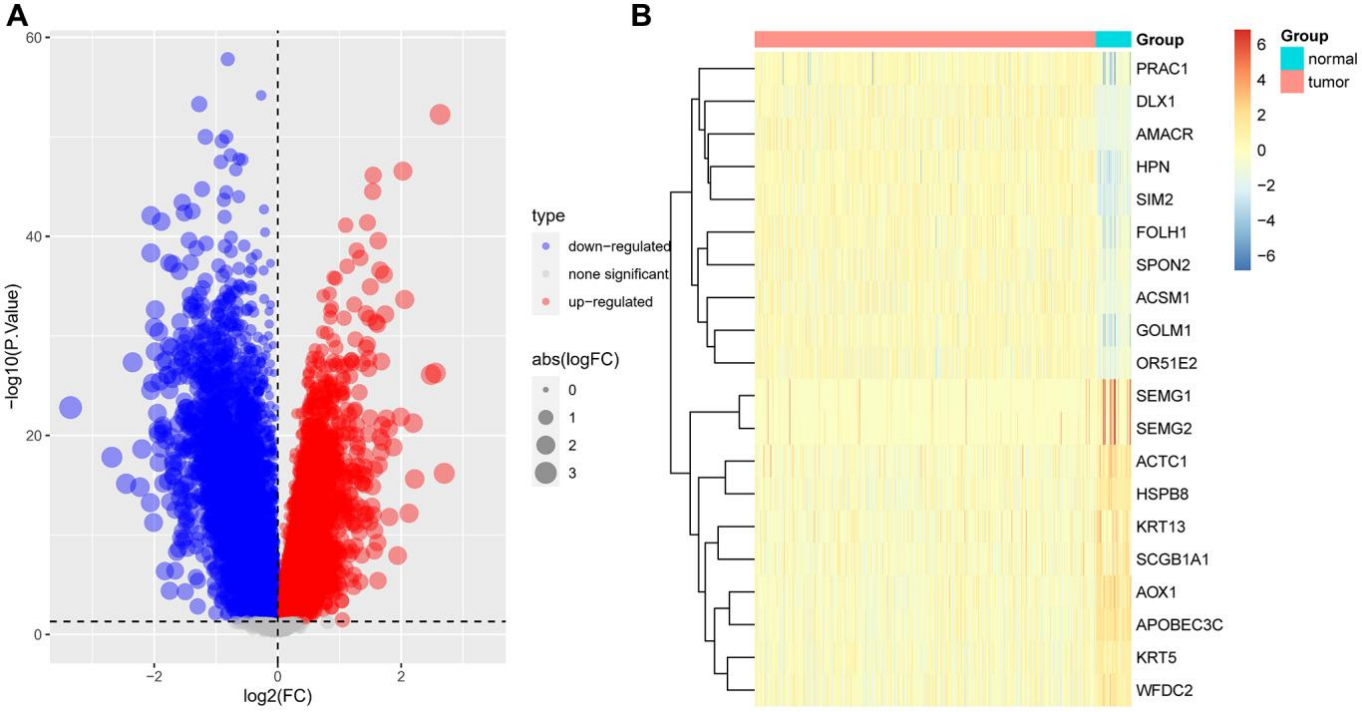
**Difference analysis.** (**A**) Volcano map displayed the differential expressed genes between tumor and normal tissues based on TCGA-PRAD data. (**B**) Heatmap displayed the expression of top 10 highly and lowly expressed genes.

**Figure 6:**
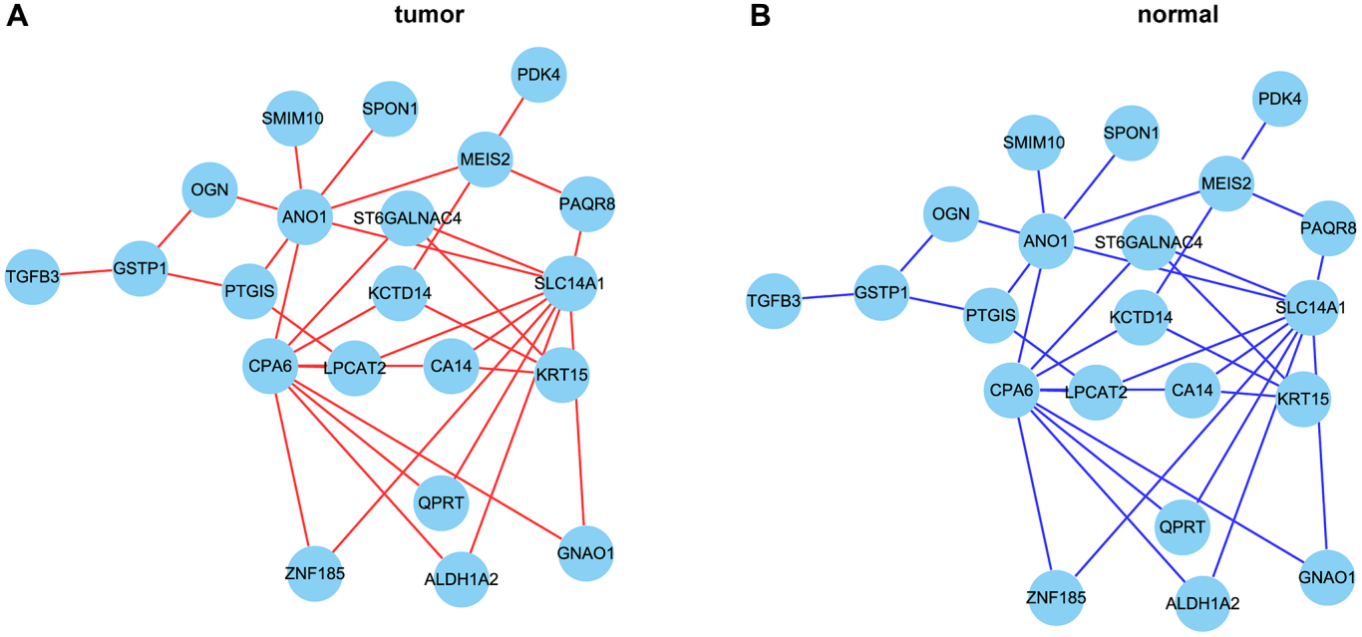
**The correlation network of hub genes.** (**A**, **B**) The correlation network of hub genes in tumor (**A**) and normal (**B**) tissues. Red lines represent positive correlation and blue lines represent negative correlation.

In order to further narrow the gene range, we conducted differential analysis to screen genes differentially expressed between tumor and normal tissues. A total of 518 differentially expressed genes were screened (Figure 5A, 5B). Through intersection, we finally got 33 gene pairs, including 21 genes, as hub genes associated with prostate cancer (Figure 6A, 6B). For example, ALDH1A2 was positively correlated with CPA6 (r = 0.5, *p* < 0.0001) in tumor tissues, while in normal tissues, ALDH1A2 expression was negative (r = −0.63, *p* < 0.0001) correlated with CPA6 (Table 1).

**Table 1:** The 33 gene pairs associated with prostate cancer.

**molecule.X**	**molecule.Y**	**r1**	**p1**	**r2**	**p2**	**FDR**	**molecule.X.type**	**molecule.Y.type**
ALDH1A2	CPA6	0.50560432	9.68E-34	−0.625984519	6.95E-07	3.50E-12	down-regulated	down-regulated
ALDH1A2	SLC14A1	0.586365345	2.06E-47	−0.511834677	0.000105168	3.50E-12	down-regulated	down-regulated
ANO1	CPA6	0.547409474	2.36E-40	−0.59298223	3.62E-06	3.50E-12	down-regulated	down-regulated
ANO1	MEIS2	0.746567384	5.28E-90	−0.525289722	6.38E-05	3.50E-12	down-regulated	down-regulated
ANO1	OGN	0.625530891	1.52E-55	−0.575241071	8.17E-06	3.50E-12	down-regulated	down-regulated
ANO1	PTGIS	0.660572094	7.20E-64	−0.561343956	1.50E-05	3.50E-12	down-regulated	down-regulated
ANO1	SLC14A1	0.606544668	1.84E-51	−0.59223007	3.75E-06	3.50E-12	down-regulated	down-regulated
ANO1	SMIM10	0.652452715	7.67E-62	−0.501931563	0.000149985	3.50E-12	down-regulated	down-regulated
ANO1	SPON1	0.683074099	7.81E-70	−0.530235037	5.28E-05	3.50E-12	down-regulated	down-regulated
CA14	CPA6	0.531974994	8.36E-38	−0.595818979	3.16E-06	3.50E-12	down-regulated	down-regulated
CA14	KRT15	0.53691886	1.32E-38	−0.598971236	2.72E-06	3.50E-12	down-regulated	down-regulated
CA14	SLC14A1	0.559469551	1.93E-42	−0.569626734	1.05E-05	3.50E-12	down-regulated	down-regulated
CPA6	GNAO1	0.529049644	2.46E-37	−0.657029628	1.22E-07	3.50E-12	down-regulated	down-regulated
CPA6	KCTD14	0.541932195	1.96E-39	−0.604657398	2.06E-06	3.50E-12	down-regulated	down-regulated
CPA6	LPCAT2	0.563398826	3.88E-43	−0.585255042	5.19E-06	3.50E-12	down-regulated	down-regulated
CPA6	QPRT	0.558227102	3.20E-42	−0.594808843	3.32E-06	3.50E-12	down-regulated	down-regulated
CPA6	ST6GALNAC4	0.588413349	8.23E-48	−0.563392132	1.37E-05	3.50E-12	down-regulated	down-regulated
CPA6	ZNF185	0.620624099	1.84E-54	−0.559644258	1.61E-05	3.50E-12	down-regulated	down-regulated
GNAO1	SLC14A1	0.590343745	3.45E-48	−0.592675755	3.67E-06	3.50E-12	down-regulated	down-regulated
GSTP1	OGN	0.557692221	3.98E-42	−0.521134666	7.46E-05	1.12E-11	down-regulated	down-regulated
GSTP1	PTGIS	0.560032479	1.54E-42	−0.533082911	4.73E-05	3.50E-12	down-regulated	down-regulated
GSTP1	TGFB3	0.501432929	3.95E-33	−0.521626064	7.32E-05	2.54E-10	down-regulated	down-regulated
KCTD14	KRT15	0.587082984	1.49E-47	−0.586132826	4.98E-06	3.50E-12	down-regulated	down-regulated
KCTD14	MEIS2	0.61606687	1.79E-53	−0.538377546	3.84E-05	3.50E-12	down-regulated	down-regulated
KRT15	ST6GALNAC4	0.536590151	1.49E-38	−0.622358865	8.40E-07	3.50E-12	down-regulated	down-regulated
LPCAT2	PTGIS	0.523149309	2.10E-36	−0.514038498	9.70E-05	1.12E-10	down-regulated	down-regulated
LPCAT2	SLC14A1	0.602645344	1.17E-50	−0.577844265	7.27E-06	3.50E-12	down-regulated	down-regulated
MEIS2	PAQR8	0.647753747	1.07E-60	−0.526524919	6.09E-05	3.50E-12	down-regulated	down-regulated
MEIS2	PDK4	0.506870988	6.29E-34	−0.517765939	8.46E-05	2.20E-10	down-regulated	down-regulated
PAQR8	SLC14A1	0.548209239	1.73E-40	−0.62366366	7.85E-07	3.50E-12	down-regulated	down-regulated
QPRT	SLC14A1	0.58784082	1.06E-47	−0.536428175	4.15E-05	3.50E-12	down-regulated	down-regulated
SLC14A1	ST6GALNAC4	0.564645996	2.32E-43	−0.584672632	5.33E-06	3.50E-12	down-regulated	down-regulated
SLC14A1	ZNF185	0.669184505	4.33E-66	−0.575177683	8.19E-06	3.50E-12	down-regulated	down-regulated

### Comprehensive analysis of hub genes in prostate cancer

**Figure 7:**
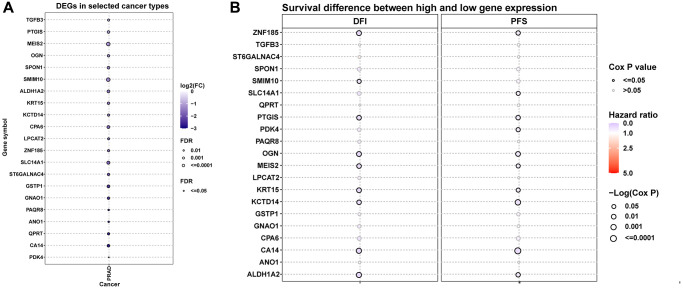
**Differential expression and prognostic value of hub genes.** (**A**) The differential expression of hub genes in TCGA-PRAD. (**B**) The prognostic value of hub genes in TCGA-PRAD, including DFI and PFS.

We next conducted comprehensive analysis of hub genes in prostate cancer. Figure 7A displayed the differential expression of these genes. Results indicated that all these genes were down-regulated in tumor tissues. The prognostic value of hub genes in prostate cancer was analyzed (Figure 7B). For example, patients with high expression of ZNF185 have better DFI (Disease Free Interval) and PFS (Progression Free Survival) in prostate cancer.

**Figure 8:**
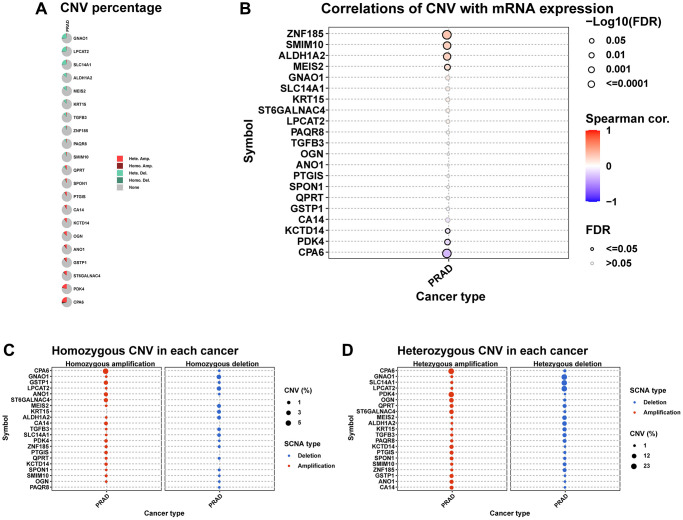
**The CNV of hub genes.** (**A**) Pie plot summarizes the CNV of hub genes in TCGA-PRAD. (**B**) The correlation between CNV and mRNA expression of hub genes in TCGA-PRAD. (**C**) Figure provides the profile of homozygous CNV of hub genes in TCGA-PRAD. (**D**) Figure provides the profile of heterozygous CNV of imputed genes in TCGA-PRAD.

**Figure 9:**
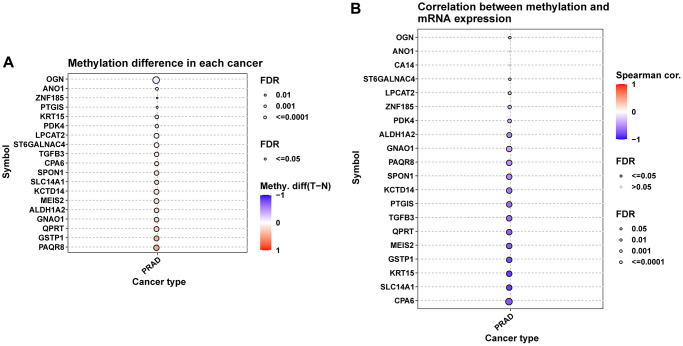
**Methylation analysis of hub genes.** (**A**) Figure summarizes the methylation difference between tumor and normal samples of hub genes in TCGA-PRAD. (**B**) Figure summarizes the correlation of methylation level with their mRNA expression TCGA-PRAD.

We further explored the CNV, mutation, and methylation level of hub genes in prostate cancer. All these genes have relatively lower mutation frequency in prostate cancer (Supplementary Figure 1A, 1B). CPA6 has highest amplification frequency (Figure 8A). The relationship between CNV and mRNA expression indicated that ZNF15, SMIM10, ALDH1A2, and MEIS2 expression were positively correlated with their CNV level (Figure 8B). Figure 8C, 8D provided the profile of homozygous and heterozygous CNV of hub genes in prostate cancer. The results of methylation analysis indicated that the methylation level of these genes were generally up-regulated in tumor prostate tissues compared to normal tissues (Figure 9A). For example, the methylation level of CPA6 was higher in tumor tissues. In addition, methylation level of CPA6 was negatively linked to its mRNA expression (Figure 9B).

### Immune infiltration analysis

**Figure 10:**
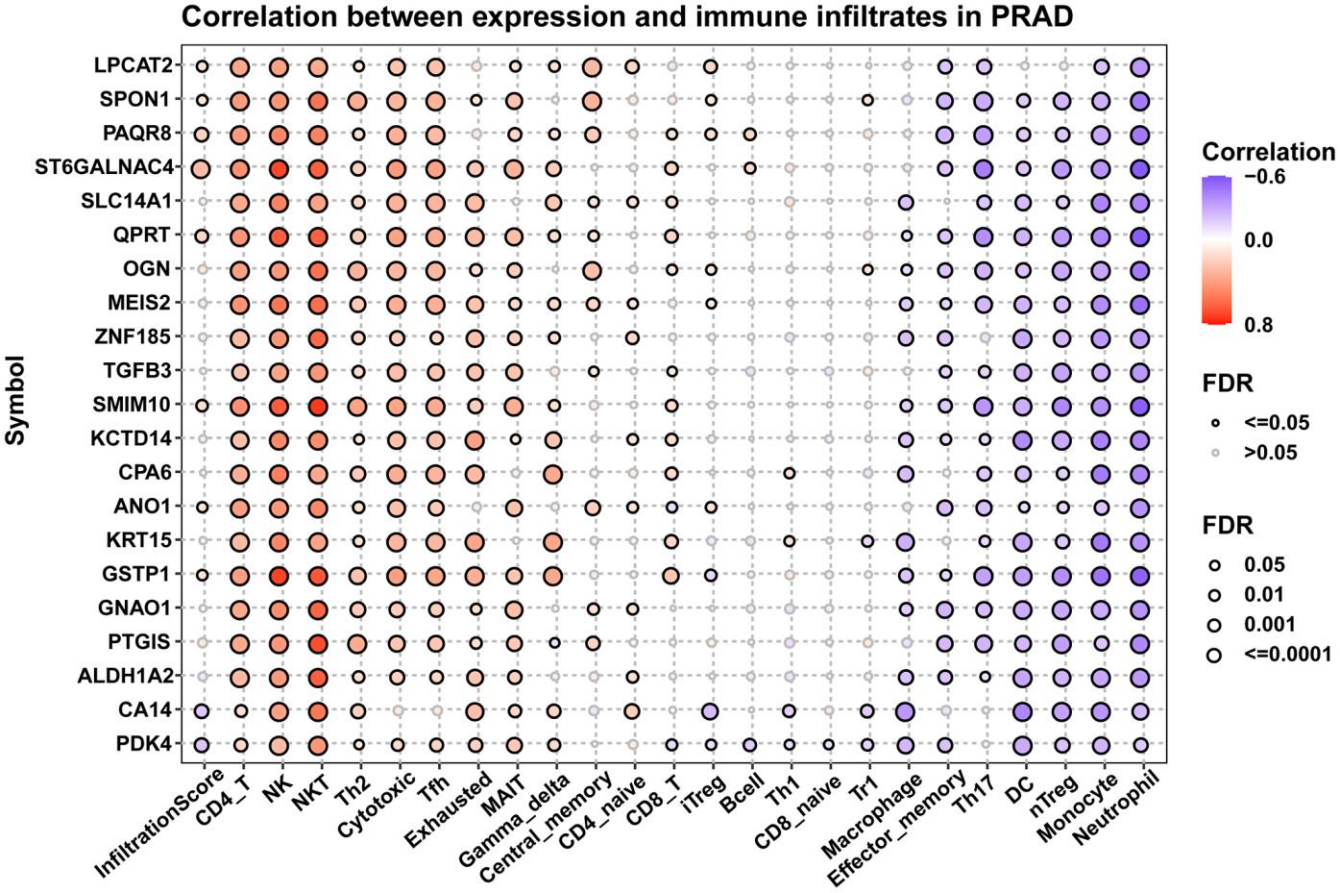
**Immune infiltration analysis.** Figure displayed the correlation of hub genes with infiltration levels of indicated immune cells.

Since immune cells in the tumor microenvironment are important factors affecting the prognosis of tumor patients, we analyzed the correlation between 21 hub genes and immune cell infiltration (Figure 10). Results suggested that these genes were positively associated with most immune cells, such as CD4 T cells, NK cells, NKT cells, and Th2 cells. These results provided evidence that high expression of these genes predicted better prognosis of prostate cancer patients.

### Drug resistance analysis

**Figure 11:**
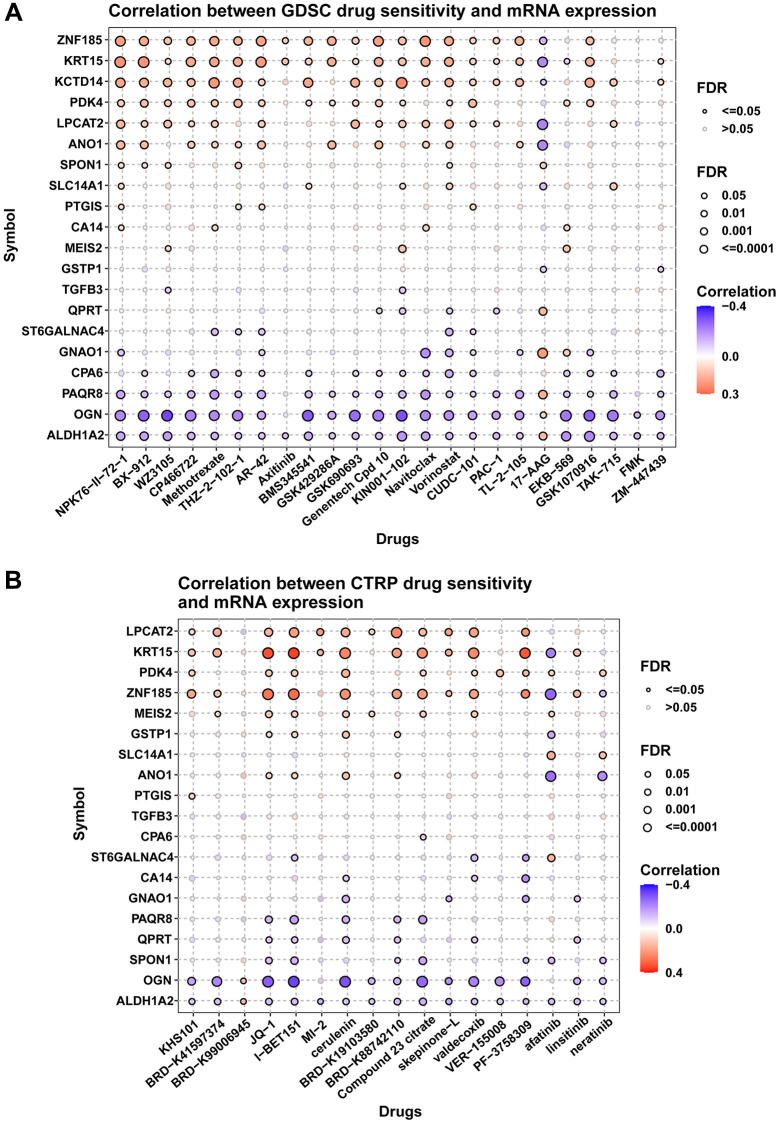
**Drug resistance analysis.** (**A**) Figure summarizes the correlation between gene expression and the sensitivity of GDSC drugs. (**B**) Figure summarizes the correlation between gene expression and the sensitivity of CTRP drugs. Pearson correlation analysis was performed to get the correlation between gene mRNA expression and drug IC50. *P*-value was adjusted by FDR.

We also explored the influence of these genes on resistance of anti-tumor drugs. Figure summarizes the correlation between gene expression and the sensitivity of GDSC drugs using GSCA database. Figure 11A–11B respectively summarized the correlation of gene expression with the sensitivity of GDSC and CTRP drugs. For example, patients with high expression of ZNF185 were resistant to AR-42 (a HDAC inhibitor) treatment, while sensitive to 17-AAG (a HSP90 inhibitor) treatment based on GDSC data.

### ALDH1A2-Drug resistance correlation

The ALDH1A2-CPA6 pair showed the most significant correlation reversal (tumor: r = +0.51 vs. normal: r = −0.63, FDR = 3.5 × 10^−^¹²). Functional analysis revealed:

- Strong association with docetaxel resistance (r = 0.43, *p* = 0.002) in TCGA cohort
- Enrichment in oxidative stress response pathways (GO:0006979, FDR = 1.2 × 10^−8^)
- Co-expression with ABC transporters (ABCB1 r = 0.38, *p* = 0.007).

## DISCUSSION

In recent years, new therapies as well as the application of next-generation sequencing to prostate cancer have changed the landscape of prostate cancer treatment [16–18]. Prostate cancer is one of the most common cancers in men, and although most patients do not have a long disease course and pose less threat to death, many still develop into intermediate-or high-risk locally advanced or metastatic cancer [19–21]. At present, the pathogenesis of prostate cancer remains unclear, and better tumor markers are lacking. Therefore, we urgently need to explore new pathogenesis as well as prognostic markers for prostate cancer.

With the development of bioinformatics, multiple methods were used to identify tumor biomarkers [22, 23]. Previous works have focused on exploring gene interaction networks constructed from a series of genes with related. Investigators always tend to ignore the impact of different states on the correlation and the reasons behind it, such as contrasting the differences in gene correlations in normal and tumor tissues. This study focuses on comparing the differential gene correlations between tumor and normal tissues in prostate cancer, and constructs a gene correlation network. The candidate genes and their target genes in the gene correlation network can be further used for the experimental study of biological functions, thereby guiding the diagnosis, treatment, and predicting prognosis of patients.

In our study, we first conducted WGCNA in TCGA-PRAD to screen key gene modules associated with prostate cancer. MEplum1, MEblue, and MEmediumpurple3 gene modules (*p* < 0.05, r > 0.3) were selected for subsequent analysis. GO and KEGG analyzes were further performed to explore the function of each gene module. Results indicated that these genes in MEblue were enriched in external encapsulating structure organization and extracellular matrix organization in BP terms. Functional enrichment analysis revealed that MEblue module genes were significantly associated with: External encapsulating structure organization (GO:0045229, FDR = 3.2 × 10^−6^): Refers to the assembly of basement membrane components (laminins, collagens IV) surrounding prostate glands, a process disrupted during tumor invasion [24]. Extracellular matrix (ECM) reorganization (GO:0030198, FDR = 1.8 × 10^−8^): Involves MMP-mediated ECM remodeling characteristics of metastatic progression [25]. Collagen-containing extracellular matrix and cell-cell junction in CC terms, extracellular matrix structural constituent and glycosaminoglycan binding in MF terms, and PI3K-Akt signaling pathway in KEGG terms. These results indicated that these genes were closely associated various malignant pathways. The genes in the MEplum1, MEblue, and MEmediumpurple3 modules were further selected to assess differential correlations via R package “DiffCorr”. A total of 313 gene pairs of gene were screened. Through intersecting with DEGs, we finally got 33 gene pairs, including 21 genes, as hub genes associated with prostate cancer. For example, ALDH1A2 was positively correlated with CPA6 (r = 0.5, *p* < 0.0001) in tumor tissues, while in normal tissues, ALDH1A2 expression was negative (r = −0.63, *p* < 0.0001) correlated with CPA6. We also conducted comprehensive analysis of hub genes in prostate cancer, such as CNV, mutation, and methylation.

Since immune cells in the tumor microenvironment are important factors affecting the prognosis of tumor patients [26], we analyzed the correlation between 21 hub genes and immune cell infiltration. Results suggested that these genes were positively associated with most immune cells, such as CD4 T cells, NK cells, NKT cells, and Th2 cells. These results provided evidence that high expression of these genes predicted better prognosis of prostate cancer patients.

We also explored the influence of these genes on resistance of anti-tumor drugs. For example, patients with high expression of ZNF185 were resistant to AR-42 (a HDAC inhibitor) treatment, while sensitive to 17-AAG (a HSP90 inhibitor) treatment based on GDSC data. These results may provide a reference for patients to choose medication. Three key limitations warrant consideration: (1) TCGA’s ethnic homogeneity may limit generalizability, (2) bulk sequencing could mask cell-type specific interactions, and (3) functional validation is needed for the 5 novel biomarkers (e.g., SMIM10). Priority next steps include single-cell validation of the ALDH1A2-CPA6 axis and testing these biomarkers in liquid biopsy cohorts.

## CONCLUSIONS

In conclusion, our study identified 21 hub genes and their potential function involved in prostate cancer. Our work provides a new direction for future research to investigate the underlying mechanism of prostate cancer.

## Supplementary Materials

Supplementary Figure 1

Supplementary Table 1
