# Supplementary material for: Identification of key genes with differential correlations in prostate cancer
Source: Aging (Albany NY). 2025 Oct 10;17(10):2582–97. doi: 10.18632/aging.206323 (PMC12606966; doi:10.18632/aging.206323)
Supplement: Supplementary Figure 1 [file aging-17-10-206323-s001.pdf]

SUPPLEMENTARY FIGURE

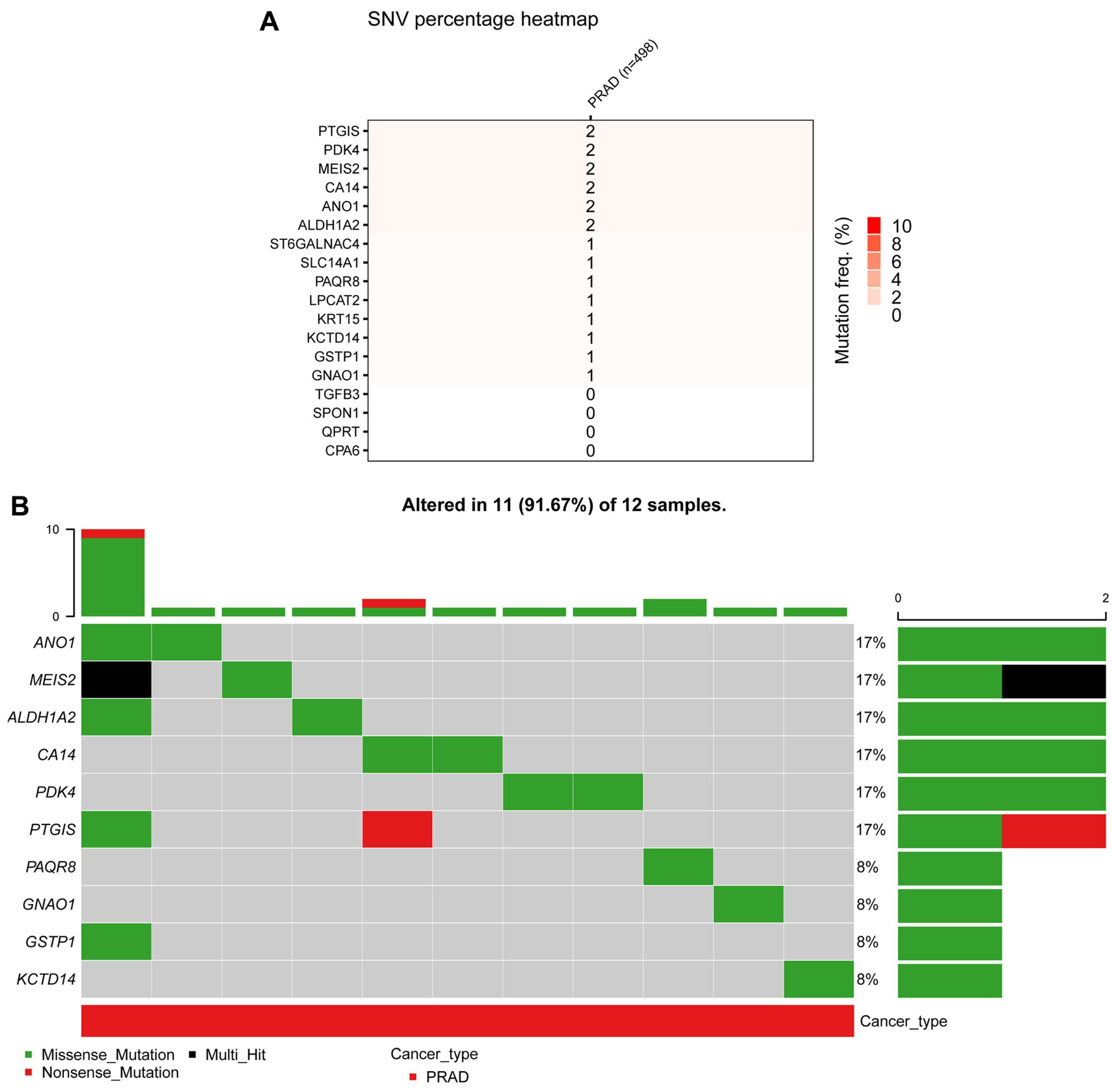

Supplementary Figure 1. Gene mutation analysis. (**A**) Figure summarizes the frequency of deleterious mutations in TCGA-PRAD. (**B**) Figure displayed the mutation information of indicated genes in TCGA-PRAD.
