# Supplementary material for: Identification of key genes with differential correlations in prostate cancer
Source: Aging (Albany NY). 2025 Oct 10;17(10):2582–97. doi: 10.18632/aging.206323 (PMC12606966; doi:10.18632/aging.206323)
Supplement: Supplementary Table 1 [file aging-17-10-206323-s002.docx]

**Supplementary Table 1. The gene pairs in MEblue module and MEmediumpurple3.**

| molecule.X | molecule.Y | r1 | p1 | r2 | p2 | FDR | Module |  |
| --- | --- | --- | --- | --- | --- | --- | --- | --- |
| A4GALT | CDC42EP5 | 0.506048048 | 8.33E-34 | -0.553990936 | 2.04E-05 | 2.49E-11 | MEblue |  |
| A4GALT | NBL1 | 0.561712227 | 7.75E-43 | -0.667381775 | 6.54E-08 | 3.50E-12 | MEblue |  |
| ABCC3 | ANKRD35 | 0.532079872 | 8.04E-38 | -0.53747053 | 3.98E-05 | 1.84E-11 | MEblue |  |
| ABCC3 | MEIS2 | 0.61669449 | 1.31E-53 | -0.519205424 | 8.02E-05 | 3.50E-12 | MEblue |  |
| ABCC3 | PPM1M | 0.507461889 | 5.14E-34 | -0.569980179 | 1.03E-05 | 1.12E-11 | MEblue |  |
| ABCC3 | SOX2 | 0.500387353 | 5.61E-33 | -0.508406468 | 0.000119066 | 4.58E-10 | MEblue |  |
| ABCG2 | CRISPLD2 | 0.510183574 | 2.02E-34 | -0.60589101 | 1.94E-06 | 3.50E-12 | MEblue |  |
| ACSF2 | C1R | 0.54533539 | 5.29E-40 | -0.513145902 | 0.000100257 | 3.00E-11 | MEblue |  |
| ACSF2 | FOXF1 | 0.505266329 | 1.09E-33 | -0.555408423 | 1.92E-05 | 2.49E-11 | MEblue |  |
| ACSF2 | PLEKHO1 | 0.576374416 | 1.64E-45 | -0.503408053 | 0.000142351 | 1.12E-11 | MEblue |  |
| ACSF2 | PRRX2 | 0.603473758 | 7.94E-51 | -0.506896905 | 0.000125702 | 3.50E-12 | MEblue |  |
| ACSF2 | RPL39L | 0.511398353 | 1.33E-34 | -0.549092655 | 2.50E-05 | 2.49E-11 | MEblue |  |
| AJAP1 | EOGT | 0.584241495 | 5.28E-47 | -0.537000151 | 4.06E-05 | 3.50E-12 | MEblue |  |
| AJAP1 | FBXO17 | 0.51488829 | 3.94E-35 | -0.562601664 | 1.42E-05 | 1.12E-11 | MEblue |  |
| AJAP1 | FOXF1 | 0.556117775 | 7.50E-42 | -0.55544462 | 1.92E-05 | 3.50E-12 | MEblue |  |
| AJAP1 | MEIS2 | 0.640232511 | 6.63E-59 | -0.652792449 | 1.57E-07 | 3.50E-12 | MEblue |  |
| AJAP1 | MKX | 0.550102284 | 8.21E-41 | -0.536698572 | 4.10E-05 | 8.40E-12 | MEblue |  |
| AJAP1 | NTN1 | 0.522210751 | 2.94E-36 | -0.543059466 | 3.19E-05 | 2.06E-11 | MEblue |  |
| AJAP1 | PDE4D | 0.514757399 | 4.13E-35 | -0.542388756 | 3.27E-05 | 4.40E-11 | MEblue |  |
| AJAP1 | PRNP | 0.532294329 | 7.43E-38 | -0.582029395 | 6.02E-06 | 3.50E-12 | MEblue |  |
| AJAP1 | RARB | 0.556286278 | 7.01E-42 | -0.631106947 | 5.28E-07 | 3.50E-12 | MEblue |  |
| AJAP1 | SLC16A2 | 0.58749305 | 1.24E-47 | -0.581644919 | 6.12E-06 | 3.50E-12 | MEblue |  |
| AJAP1 | SMIM10 | 0.544910541 | 6.23E-40 | -0.519225688 | 8.01E-05 | 2.06E-11 | MEblue |  |
| AJAP1 | TMEM106A | 0.534443919 | 3.34E-38 | -0.571674187 | 9.57E-06 | 3.50E-12 | MEblue |  |
| AJAP1 | TSHZ3 | 0.531411997 | 1.03E-37 | -0.588437828 | 4.48E-06 | 3.50E-12 | MEblue |  |
| AJAP1 | TWIST2 | 0.503793904 | 1.79E-33 | -0.505646493 | 0.000131453 | 4.58E-10 | MEblue |  |
| AJAP1 | WWTR1 | 0.550126915 | 8.13E-41 | -0.50730227 | 0.000123888 | 4.40E-11 | MEblue |  |
| ALDH1A2 | CPA6 | 0.50560432 | 9.68E-34 | -0.625984519 | 6.95E-07 | 3.50E-12 | MEblue |  |
| ALDH1A2 | SLC14A1 | 0.586365345 | 2.06E-47 | -0.511834677 | 0.000105168 | 3.50E-12 | MEblue |  |
| AMOT | ARL4C | 0.570177375 | 2.30E-44 | -0.511400972 | 0.000106841 | 1.12E-11 | MEblue |  |
| AMOT | MEIS2 | 0.544091596 | 8.55E-40 | -0.504939048 | 0.000134812 | 5.40E-11 | MEblue |  |
| ANGPTL2 | ANO1 | 0.679424403 | 7.88E-69 | -0.615337649 | 1.21E-06 | 3.50E-12 | MEblue |  |
| ANGPTL2 | C11orf45 | 0.512928698 | 7.81E-35 | -0.649600294 | 1.89E-07 | 3.50E-12 | MEblue |  |
| ANGPTL2 | LPCAT2 | 0.607639069 | 1.09E-51 | -0.575275585 | 8.16E-06 | 3.50E-12 | MEblue |  |
| ANGPTL2 | ST6GALNAC4 | 0.578153533 | 7.60E-46 | -0.535390307 | 4.32E-05 | 3.50E-12 | MEblue |  |
| ANGPTL2 | TLR2 | 0.5082249 | 3.96E-34 | -0.505933427 | 0.000130113 | 3.81E-10 | MEblue |  |
| ANGPTL2 | ZFP92 | 0.568210655 | 5.25E-44 | -0.552675045 | 2.15E-05 | 3.50E-12 | MEblue |  |
| ANKRD35 | ANO1 | 0.554102389 | 1.68E-41 | -0.562799909 | 1.41E-05 | 3.50E-12 | MEblue |  |
| ANKRD35 | C11orf45 | 0.628941984 | 2.62E-56 | -0.542793901 | 3.22E-05 | 3.50E-12 | MEblue |  |
| ANKRD35 | HOXB2 | 0.59669838 | 1.89E-49 | -0.540884455 | 3.48E-05 | 3.50E-12 | MEblue |  |
| molecule.X | molecule.Y | r1 | p1 | r2 | p2 | FDR | Module |  |
| ANKRD35 | KCTD14 | 0.585950021 | 2.48E-47 | -0.538130399 | 3.88E-05 | 3.50E-12 | MEblue |  |
| ANKRD35 | PAQR7 | 0.559442402 | 1.96E-42 | -0.528395667 | 5.67E-05 | 8.40E-12 | MEblue |  |
| ANKRD35 | RASL11B | 0.525034313 | 1.06E-36 | -0.5042158 | 0.000138327 | 1.56E-10 | MEblue |  |
| ANKRD35 | VPS37C | -0.523456589 | 1.88E-36 | 0.501726389 | 0.000151075 | 2.20E-10 | MEblue |  |
| ANO1 | ARL4C | 0.67936613 | 8.17E-69 | -0.582714643 | 5.83E-06 | 3.50E-12 | MEblue |  |
| ANO1 | C1R | 0.612338701 | 1.12E-52 | -0.578937487 | 6.92E-06 | 3.50E-12 | MEblue |  |
| ANO1 | C1S | 0.665354509 | 4.30E-65 | -0.544917377 | 2.96E-05 | 3.50E-12 | MEblue |  |
| ANO1 | CDH3 | 0.55870781 | 2.64E-42 | -0.513496603 | 9.90E-05 | 1.47E-11 | MEblue |  |
| ANO1 | CPA6 | 0.547409474 | 2.36E-40 | -0.59298223 | 3.62E-06 | 3.50E-12 | MEblue |  |
| ANO1 | CRISPLD2 | 0.614056034 | 4.82E-53 | -0.653676159 | 1.49E-07 | 3.50E-12 | MEblue |  |
| ANO1 | DPY19L1 | 0.630169041 | 1.38E-56 | -0.509908545 | 0.000112782 | 3.50E-12 | MEblue |  |
| ANO1 | EPHA3 | 0.705963066 | 1.76E-76 | -0.620802174 | 9.12E-07 | 3.50E-12 | MEblue |  |
| ANO1 | FEZ2 | 0.513604838 | 6.17E-35 | -0.620084672 | 9.46E-07 | 3.50E-12 | MEblue |  |
| ANO1 | FOXF1 | 0.649195481 | 4.80E-61 | -0.616978467 | 1.11E-06 | 3.50E-12 | MEblue |  |
| ANO1 | GPD1L | 0.522469666 | 2.68E-36 | -0.512946646 | 0.00010099 | 1.12E-10 | MEblue |  |
| ANO1 | KCTD9 | 0.557467306 | 4.35E-42 | -0.662975611 | 8.56E-08 | 3.50E-12 | MEblue |  |
| ANO1 | KDELC2 | 0.621545111 | 1.15E-54 | -0.568443724 | 1.10E-05 | 3.50E-12 | MEblue |  |
| ANO1 | LRCH2 | 0.513008423 | 7.60E-35 | -0.525943995 | 6.22E-05 | 1.11E-10 | MEblue |  |
| ANO1 | LUM | 0.505649968 | 9.53E-34 | -0.566955354 | 1.18E-05 | 1.47E-11 | MEblue |  |
| ANO1 | MEIS2 | 0.746567384 | 5.28E-90 | -0.525289722 | 6.38E-05 | 3.50E-12 | MEblue |  |
| ANO1 | MFAP2 | 0.570616158 | 1.91E-44 | -0.551295071 | 2.28E-05 | 3.50E-12 | MEblue |  |
| ANO1 | MFAP4 | 0.619781459 | 2.81E-54 | -0.589246072 | 4.31E-06 | 3.50E-12 | MEblue |  |
| ANO1 | MKX | 0.680046607 | 5.33E-69 | -0.621245296 | 8.91E-07 | 3.50E-12 | MEblue |  |
| ANO1 | NPTN | 0.606178456 | 2.19E-51 | -0.644257353 | 2.56E-07 | 3.50E-12 | MEblue |  |
| ANO1 | OGN | 0.625530891 | 1.52E-55 | -0.575241071 | 8.17E-06 | 3.50E-12 | MEblue |  |
| ANO1 | PLB1 | 0.523303003 | 1.99E-36 | -0.515544125 | 9.18E-05 | 1.12E-10 | MEblue |  |
| ANO1 | PPM1M | 0.508910623 | 3.13E-34 | -0.58262437 | 5.85E-06 | 3.50E-12 | MEblue |  |
| ANO1 | PRNP | 0.683701018 | 5.23E-70 | -0.535141786 | 4.36E-05 | 3.50E-12 | MEblue |  |
| ANO1 | PRRX2 | 0.526044997 | 7.37E-37 | -0.652411674 | 1.60E-07 | 3.50E-12 | MEblue |  |
| ANO1 | PTGER2 | 0.682092439 | 1.46E-69 | -0.612797093 | 1.37E-06 | 3.50E-12 | MEblue |  |
| ANO1 | PTGIS | 0.660572094 | 7.20E-64 | -0.561343956 | 1.50E-05 | 3.50E-12 | MEblue |  |
| ANO1 | PXDC1 | 0.508431183 | 3.69E-34 | -0.658768203 | 1.10E-07 | 3.50E-12 | MEblue |  |
| ANO1 | RARB | 0.731264816 | 1.29E-84 | -0.510079372 | 0.000112087 | 3.50E-12 | MEblue |  |
| ANO1 | SEPT7 | 0.56874788 | 4.19E-44 | -0.523742631 | 6.76E-05 | 3.50E-12 | MEblue |  |
| ANO1 | SLC14A1 | 0.606544668 | 1.84E-51 | -0.59223007 | 3.75E-06 | 3.50E-12 | MEblue |  |
| ANO1 | SMIM10 | 0.652452715 | 7.67E-62 | -0.501931563 | 0.000149985 | 3.50E-12 | MEblue |  |
| ANO1 | SPON1 | 0.683074099 | 7.81E-70 | -0.530235037 | 5.28E-05 | 3.50E-12 | MEblue |  |
| ANO1 | THSD4 | 0.642044919 | 2.48E-59 | -0.553401965 | 2.09E-05 | 3.50E-12 | MEblue |  |
| ANO1 | TMEM237 | 0.584320025 | 5.10E-47 | -0.562836613 | 1.40E-05 | 3.50E-12 | MEblue |  |
| ANO1 | TMEM43 | 0.720810221 | 3.81E-81 | -0.535189672 | 4.35E-05 | 3.50E-12 | MEblue |  |
| ANO1 | TNC | 0.694222739 | 5.41E-73 | -0.513552329 | 9.88E-05 | 3.50E-12 | MEblue |  |
| molecule.X | molecule.Y | r1 | p1 | r2 | p2 | FDR | Module |  |
| ANO1 | TSHZ3 | 0.68368496 | 5.29E-70 | -0.518985601 | 8.08E-05 | 3.50E-12 | MEblue |  |
| ANO1 | TTC39B | 0.50908786 | 2.95E-34 | -0.531316259 | 5.06E-05 | 9.31E-11 | MEblue |  |
| ANO1 | UST | 0.557420889 | 4.44E-42 | -0.682389526 | 2.53E-08 | 3.50E-12 | MEblue |  |
| ANO1 | WWTR1 | 0.669867068 | 2.86E-66 | -0.533011969 | 4.74E-05 | 3.50E-12 | MEblue |  |
| ARHGEF40 | CRISPLD2 | 0.615925113 | 1.92E-53 | -0.501915093 | 0.000150072 | 3.50E-12 | MEblue |  |
| ARHGEF40 | EFNB3 | 0.50329671 | 2.11E-33 | -0.62239791 | 8.39E-07 | 3.50E-12 | MEblue |  |
| ARHGEF40 | SLC14A1 | 0.55647443 | 6.50E-42 | -0.511915405 | 0.00010486 | 1.84E-11 | MEblue |  |
| ARHGEF40 | TMEM237 | 0.530823255 | 1.28E-37 | -0.52113848 | 7.46E-05 | 5.37E-11 | MEblue |  |
| ARL4C | C11orf45 | 0.685887065 | 1.29E-70 | -0.541597682 | 3.38E-05 | 3.50E-12 | MEblue |  |
| ARL4C | CLU | 0.503192442 | 2.19E-33 | -0.562500206 | 1.42E-05 | 2.06E-11 | MEblue |  |
| ARL4C | FN1 | 0.50197932 | 3.29E-33 | -0.581897206 | 6.05E-06 | 8.40E-12 | MEblue |  |
| ARL4C | GNAO1 | 0.514608037 | 4.35E-35 | -0.553105893 | 2.12E-05 | 2.06E-11 | MEblue |  |
| ARL4C | HOXB2 | 0.528920685 | 2.58E-37 | -0.623013789 | 8.12E-07 | 3.50E-12 | MEblue |  |
| ARL4C | LPCAT2 | 0.632868288 | 3.37E-57 | -0.55357997 | 2.07E-05 | 3.50E-12 | MEblue |  |
| ARL4C | PAQR8 | 0.671560636 | 1.02E-66 | -0.631191813 | 5.26E-07 | 3.50E-12 | MEblue |  |
| ARL4C | PLA2G4A | 0.558126083 | 3.34E-42 | -0.58169444 | 6.11E-06 | 3.50E-12 | MEblue |  |
| ARL4C | QPRT | 0.545347675 | 5.26E-40 | -0.542517346 | 3.26E-05 | 8.40E-12 | MEblue |  |
| ARL4C | RHOBTB2 | 0.527909986 | 3.73E-37 | -0.545855667 | 2.85E-05 | 1.47E-11 | MEblue |  |
| ARL4C | ST6GALNAC4 | 0.647918991 | 9.77E-61 | -0.588520474 | 4.46E-06 | 3.50E-12 | MEblue |  |
| ARL4C | TLR2 | 0.741323817 | 4.09E-88 | -0.501173637 | 0.000154047 | 3.50E-12 | MEblue |  |
| ARL4C | WWC3 | 0.562016123 | 6.84E-43 | -0.601657272 | 2.39E-06 | 3.50E-12 | MEblue |  |
| ARL4C | ZFP92 | 0.510551837 | 1.78E-34 | -0.653377652 | 1.51E-07 | 3.50E-12 | MEblue |  |
| ARSJ | PLA2G4A | 0.544494683 | 7.32E-40 | -0.599181908 | 2.69E-06 | 3.50E-12 | MEblue |  |
| ARSJ | WWC3 | 0.585398842 | 3.16E-47 | -0.633199016 | 4.72E-07 | 3.50E-12 | MEblue |  |
| BCL11A | PAQR8 | 0.505877272 | 8.82E-34 | -0.553390216 | 2.09E-05 | 2.49E-11 | MEblue |  |
| BCL11A | VPS37C | -0.50480325 | 1.27E-33 | 0.550950521 | 2.31E-05 | 4.40E-11 | MEblue |  |
| C11orf45 | CDC42EP3 | 0.52876599 | 2.73E-37 | -0.507004679 | 0.000125218 | 1.12E-10 | MEblue |  |
| C11orf45 | FAM92A1 | 0.502663828 | 2.61E-33 | -0.531387905 | 5.05E-05 | 1.43E-10 | MEblue |  |
| C11orf45 | GNAI2 | 0.538432236 | 7.44E-39 | -0.505657176 | 0.000131403 | 6.89E-11 | MEblue |  |
| C11orf45 | IL33 | 0.508471593 | 3.64E-34 | -0.501073453 | 0.000154591 | 4.58E-10 | MEblue |  |
| C11orf45 | KRT15 | 0.642664554 | 1.77E-59 | -0.522888109 | 6.98E-05 | 3.50E-12 | MEblue |  |
| C11orf45 | MEIS2 | 0.785495808 | 1.21E-105 | -0.503739538 | 0.000140687 | 3.50E-12 | MEblue |  |
| C11orf45 | PHF19 | 0.609753918 | 3.93E-52 | -0.557153655 | 1.79E-05 | 3.50E-12 | MEblue |  |
| C11orf45 | PLB1 | 0.560181514 | 1.45E-42 | -0.50704236 | 0.000125049 | 2.06E-11 | MEblue |  |
| C11orf45 | PPM1M | 0.578557516 | 6.38E-46 | -0.504176402 | 0.000138521 | 8.40E-12 | MEblue |  |
| C11orf45 | PRNP | 0.534687518 | 3.05E-38 | -0.561359795 | 1.50E-05 | 3.50E-12 | MEblue |  |
| C11orf45 | SLC16A2 | 0.593141042 | 9.68E-49 | -0.502617774 | 0.000146392 | 3.50E-12 | MEblue |  |
| C11orf45 | SMIM10 | 0.561539623 | 8.32E-43 | -0.511725096 | 0.000105589 | 1.47E-11 | MEblue |  |
| C11orf45 | ST8SIA1 | 0.503443592 | 2.01E-33 | -0.51331356 | 9.96E-05 | 3.39E-10 | MEblue |  |
| C11orf45 | THSD4 | 0.646579056 | 2.06E-60 | -0.518890251 | 8.11E-05 | 3.50E-12 | MEblue |  |
| C11orf45 | TMEM43 | 0.536275968 | 1.68E-38 | -0.56674976 | 1.19E-05 | 3.50E-12 | MEblue |  |
| molecule.X | molecule.Y | r1 | p1 | r2 | p2 | FDR | Module |  |
| C11orf45 | TNC | 0.551835749 | 4.14E-41 | -0.568715532 | 1.09E-05 | 3.50E-12 | MEblue |  |
| C11orf45 | TSHZ3 | 0.552823501 | 2.80E-41 | -0.55272884 | 2.15E-05 | 3.50E-12 | MEblue |  |
| C11orf45 | WIPF3 | 0.529887192 | 1.81E-37 | -0.507038131 | 0.000125068 | 1.12E-10 | MEblue |  |
| C1R | GSTP1 | 0.665677804 | 3.54E-65 | -0.57079458 | 9.94E-06 | 3.50E-12 | MEblue |  |
| C1R | HOXB2 | 0.541453807 | 2.36E-39 | -0.595846511 | 3.16E-06 | 3.50E-12 | MEblue |  |
| C1S | GSTP1 | 0.575334501 | 2.56E-45 | -0.526784077 | 6.03E-05 | 3.50E-12 | MEblue |  |
| C1S | HOXB2 | 0.525332536 | 9.54E-37 | -0.584666405 | 5.33E-06 | 3.50E-12 | MEblue |  |
| C1S | KCNIP1 | 0.530631703 | 1.37E-37 | -0.524588016 | 6.55E-05 | 4.40E-11 | MEblue |  |
| CA14 | CPA6 | 0.531974994 | 8.36E-38 | -0.595818979 | 3.16E-06 | 3.50E-12 | MEblue |  |
| CA14 | KRT15 | 0.53691886 | 1.32E-38 | -0.598971236 | 2.72E-06 | 3.50E-12 | MEblue |  |
| CA14 | NTN1 | 0.519471469 | 7.82E-36 | -0.535573039 | 4.29E-05 | 4.40E-11 | MEblue |  |
| CA14 | SLC14A1 | 0.559469551 | 1.93E-42 | -0.569626734 | 1.05E-05 | 3.50E-12 | MEblue |  |
| CA14 | VSNL1 | 0.505264982 | 1.09E-33 | -0.536350321 | 4.16E-05 | 9.28E-11 | MEblue |  |
| CCDC178 | DUOX1 | 0.554242888 | 1.59E-41 | -0.501276246 | 0.000153491 | 4.40E-11 | MEblue |  |
| CCNJL | SOX2 | 0.54499317 | 6.04E-40 | -0.502568184 | 0.000146649 | 5.51E-11 | MEblue |  |
| CDCA7 | PAQR8 | 0.505799565 | 9.06E-34 | -0.547622292 | 2.65E-05 | 5.30E-11 | MEblue |  |
| CDH3 | ST6GALNAC4 | 0.532062899 | 8.10E-38 | -0.575428445 | 8.10E-06 | 3.50E-12 | MEblue |  |
| CDH3 | ZNF185 | 0.569413619 | 3.17E-44 | -0.552781437 | 2.14E-05 | 3.50E-12 | MEblue |  |
| CERK | CPA6 | 0.533146516 | 5.41E-38 | -0.552725735 | 2.15E-05 | 8.40E-12 | MEblue |  |
| CIB2 | GSTP1 | 0.503494299 | 1.98E-33 | -0.518435848 | 8.25E-05 | 2.56E-10 | MEblue |  |
| CLDN1 | TNC | 0.521557036 | 3.72E-36 | -0.500804119 | 0.000156063 | 2.55E-10 | MEblue |  |
| CLU | CRISPLD2 | 0.511113955 | 1.47E-34 | -0.536622504 | 4.12E-05 | 5.51E-11 | MEblue |  |
| CLU | NTN1 | 0.544515754 | 7.26E-40 | -0.600891622 | 2.48E-06 | 3.50E-12 | MEblue |  |
| CLU | SYNGR1 | 0.504228864 | 1.54E-33 | -0.501891062 | 0.0001502 | 5.51E-10 | MEblue |  |
| CPA6 | GNAO1 | 0.529049644 | 2.46E-37 | -0.657029628 | 1.22E-07 | 3.50E-12 | MEblue |  |
| CPA6 | KCTD14 | 0.541932195 | 1.96E-39 | -0.604657398 | 2.06E-06 | 3.50E-12 | MEblue |  |
| CPA6 | LPCAT2 | 0.563398826 | 3.88E-43 | -0.585255042 | 5.19E-06 | 3.50E-12 | MEblue |  |
| CPA6 | QPRT | 0.558227102 | 3.20E-42 | -0.594808843 | 3.32E-06 | 3.50E-12 | MEblue |  |
| CPA6 | ST6GALNAC4 | 0.588413349 | 8.23E-48 | -0.563392132 | 1.37E-05 | 3.50E-12 | MEblue |  |
| CPA6 | ZNF185 | 0.620624099 | 1.84E-54 | -0.559644258 | 1.61E-05 | 3.50E-12 | MEblue |  |
| CRISPLD2 | GSTP1 | 0.508408242 | 3.72E-34 | -0.566535357 | 1.20E-05 | 1.12E-11 | MEblue |  |
| CRISPLD2 | PDK4 | 0.535816532 | 2.00E-38 | -0.50393493 | 0.000139715 | 9.31E-11 | MEblue |  |
| CRISPLD2 | PLA2G4A | 0.500090953 | 6.19E-33 | -0.65193135 | 1.65E-07 | 3.50E-12 | MEblue |  |
| CRISPLD2 | QPRT | 0.531149519 | 1.14E-37 | -0.54808949 | 2.60E-05 | 1.12E-11 | MEblue |  |
| CRISPLD2 | ZFP92 | 0.547552291 | 2.23E-40 | -0.685116599 | 2.11E-08 | 3.50E-12 | MEblue |  |
| CRISPLD2 | ZNF185 | 0.590223139 | 3.64E-48 | -0.539073375 | 3.74E-05 | 3.50E-12 | MEblue |  |
| DCHS2 | MEIS2 | 0.667320338 | 1.33E-65 | -0.512516142 | 0.000102589 | 3.50E-12 | MEblue |  |
| DCHS2 | THSD4 | 0.503539494 | 1.95E-33 | -0.536644945 | 4.11E-05 | 9.31E-11 | MEblue |  |
| DKC1 | NBL1 | -0.541954738 | 1.95E-39 | 0.51244825 | 0.000102844 | 4.40E-11 | MEblue |  |
| DUSP15 | KRT15 | 0.566394355 | 1.12E-43 | -0.556112665 | 1.87E-05 | 3.50E-12 | MEblue |  |
| DUSP15 | NBL1 | 0.504430192 | 1.44E-33 | -0.609446295 | 1.63E-06 | 3.50E-12 | MEblue |  |
| molecule.X | molecule.Y | r1 | p1 | r2 | p2 | FDR | Module |  |
| EFNB3 | LPCAT2 | 0.501811516 | 3.48E-33 | -0.627146476 | 6.53E-07 | 3.50E-12 | MEblue |  |
| EFNB3 | PLLP | 0.502263042 | 2.99E-33 | -0.558009366 | 1.72E-05 | 2.49E-11 | MEblue |  |
| EFNB3 | ST6GALNAC4 | 0.517179373 | 1.76E-35 | -0.674434451 | 4.21E-08 | 3.50E-12 | MEblue |  |
| EIF4E3 | WWC3 | 0.556941901 | 5.38E-42 | -0.545500665 | 2.89E-05 | 3.50E-12 | MEblue |  |
| ELP3 | KCTD9 | 0.576034532 | 1.90E-45 | -0.592872799 | 3.64E-06 | 3.50E-12 | MEblue |  |
| ELP3 | LEPROTL1 | 0.684990384 | 2.29E-70 | -0.514807311 | 9.43E-05 | 3.50E-12 | MEblue |  |
| ENHO | GSTP1 | 0.541806359 | 2.06E-39 | -0.519261705 | 8.00E-05 | 2.49E-11 | MEblue |  |
| ENHO | PLLP | 0.52353367 | 1.83E-36 | -0.597711592 | 2.89E-06 | 3.50E-12 | MEblue |  |
| EPHA3 | KCNIP1 | 0.540828179 | 2.99E-39 | -0.590444497 | 4.08E-06 | 3.50E-12 | MEblue |  |
| EPHA3 | WWC3 | 0.561779008 | 7.54E-43 | -0.591941062 | 3.80E-06 | 3.50E-12 | MEblue |  |
| EYA4 | SLC14A1 | 0.525232713 | 9.90E-37 | -0.536928803 | 4.07E-05 | 2.06E-11 | MEblue |  |
| FAM124A | GATA3 | 0.500871998 | 4.77E-33 | -0.50543337 | 0.000132457 | 5.51E-10 | MEblue |  |
| FAM49A | WWC3 | 0.537182548 | 1.19E-38 | -0.551486111 | 2.26E-05 | 8.40E-12 | MEblue |  |
| FBXO17 | HOXB2 | 0.529358591 | 2.20E-37 | -0.510458512 | 0.000110559 | 1.11E-10 | MEblue |  |
| FHL2 | RASL11B | 0.514520807 | 4.48E-35 | -0.520168813 | 7.73E-05 | 1.43E-10 | MEblue |  |
| FHL2 | ZFP92 | 0.512428716 | 9.29E-35 | -0.515655205 | 9.14E-05 | 1.72E-10 | MEblue |  |
| FOXF1 | GSTP1 | 0.64845543 | 7.25E-61 | -0.534302086 | 4.51E-05 | 3.50E-12 | MEblue |  |
| FOXF1 | HOXB2 | 0.522506003 | 2.65E-36 | -0.576631721 | 7.68E-06 | 3.50E-12 | MEblue |  |
| FOXF1 | KCNIP1 | 0.546696996 | 3.12E-40 | -0.53416272 | 4.53E-05 | 1.12E-11 | MEblue |  |
| FOXF1 | LPCAT2 | 0.522459507 | 2.69E-36 | -0.57408217 | 8.60E-06 | 3.50E-12 | MEblue |  |
| GALNT14 | ST6GALNAC4 | 0.523090302 | 2.14E-36 | -0.63773593 | 3.68E-07 | 3.50E-12 | MEblue |  |
| GNAO1 | NTN1 | 0.610777787 | 2.39E-52 | -0.544395504 | 3.02E-05 | 3.50E-12 | MEblue |  |
| GNAO1 | SLC14A1 | 0.590343745 | 3.45E-48 | -0.592675755 | 3.67E-06 | 3.50E-12 | MEblue |  |
| GNAO1 | THSD4 | 0.51995726 | 6.58E-36 | -0.509453292 | 0.000114654 | 1.56E-10 | MEblue |  |
| GNAO1 | ZNF488 | 0.536639858 | 1.46E-38 | -0.568954364 | 1.08E-05 | 3.50E-12 | MEblue |  |
| GRASP | HOXB2 | 0.509858121 | 2.26E-34 | -0.543280139 | 3.16E-05 | 5.30E-11 | MEblue |  |
| GSTM3 | NBL1 | 0.525966286 | 7.58E-37 | -0.575175771 | 8.19E-06 | 3.50E-12 | MEblue |  |
| GSTP1 | NBL1 | 0.615331439 | 2.57E-53 | -0.508164617 | 0.000120108 | 3.50E-12 | MEblue |  |
| GSTP1 | OGN | 0.557692221 | 3.98E-42 | -0.521134666 | 7.46E-05 | 1.12E-11 | MEblue |  |
| GSTP1 | PRRX2 | 0.767864146 | 3.51E-98 | -0.513510652 | 9.89E-05 | 3.50E-12 | MEblue |  |
| GSTP1 | PTGER2 | 0.523760994 | 1.68E-36 | -0.607871838 | 1.76E-06 | 3.50E-12 | MEblue |  |
| GSTP1 | PTGIS | 0.560032479 | 1.54E-42 | -0.533082911 | 4.73E-05 | 3.50E-12 | MEblue |  |
| GSTP1 | RPL39L | 0.556799837 | 5.70E-42 | -0.579140528 | 6.86E-06 | 3.50E-12 | MEblue |  |
| GSTP1 | SORBS3 | 0.557428304 | 4.42E-42 | -0.510839372 | 0.000109042 | 1.84E-11 | MEblue |  |
| GSTP1 | TGFB3 | 0.501432929 | 3.95E-33 | -0.521626064 | 7.32E-05 | 2.54E-10 | MEblue |  |
| HOXB2 | HTRA1 | 0.529320974 | 2.23E-37 | -0.51671385 | 8.79E-05 | 6.89E-11 | MEblue |  |
| HOXB2 | LAYN | 0.583692349 | 6.74E-47 | -0.520749908 | 7.57E-05 | 3.50E-12 | MEblue |  |
| HOXB2 | MFAP2 | 0.523373553 | 1.94E-36 | -0.534223124 | 4.52E-05 | 4.40E-11 | MEblue |  |
| HOXB2 | NTN1 | 0.520417372 | 5.59E-36 | -0.584999907 | 5.25E-06 | 3.50E-12 | MEblue |  |
| HOXB2 | PRRX2 | 0.547950627 | 1.91E-40 | -0.604097626 | 2.12E-06 | 3.50E-12 | MEblue |  |
| HOXB2 | PTPLAD2 | 0.523839564 | 1.64E-36 | -0.522284001 | 7.14E-05 | 6.89E-11 | MEblue |  |
| molecule.X | molecule.Y | r1 | p1 | r2 | p2 | FDR | Module |  |
| HOXB2 | RARRES2 | 0.54856462 | 1.50E-40 | -0.504264659 | 0.000138087 | 5.30E-11 | MEblue |  |
| HRASLS5 | TRPV4 | 0.559313982 | 2.06E-42 | -0.508253053 | 0.000119726 | 2.06E-11 | MEblue |  |
| KCNIP1 | MEIS2 | 0.516966758 | 1.90E-35 | -0.663416906 | 8.33E-08 | 3.50E-12 | MEblue |  |
| KCNIP1 | MKX | 0.522437413 | 2.71E-36 | -0.550604785 | 2.35E-05 | 1.47E-11 | MEblue |  |
| KCNIP1 | PRNP | 0.529077967 | 2.43E-37 | -0.561223417 | 1.50E-05 | 3.50E-12 | MEblue |  |
| KCNIP1 | PTGIS | 0.56017692 | 1.45E-42 | -0.526988755 | 5.98E-05 | 8.40E-12 | MEblue |  |
| KCNIP1 | SLC16A2 | 0.571942923 | 1.09E-44 | -0.607628112 | 1.78E-06 | 3.50E-12 | MEblue |  |
| KCTD14 | KRT15 | 0.587082984 | 1.49E-47 | -0.586132826 | 4.98E-06 | 3.50E-12 | MEblue |  |
| KCTD14 | MEIS2 | 0.61606687 | 1.79E-53 | -0.538377546 | 3.84E-05 | 3.50E-12 | MEblue |  |
| KCTD14 | PDLIM4 | 0.526458449 | 6.34E-37 | -0.574133242 | 8.58E-06 | 3.50E-12 | MEblue |  |
| KCTD9 | LPCAT2 | 0.513830539 | 5.71E-35 | -0.60672459 | 1.86E-06 | 3.50E-12 | MEblue |  |
| KCTD9 | PLA2G4A | 0.56222861 | 6.27E-43 | -0.525188813 | 6.40E-05 | 8.40E-12 | MEblue |  |
| KCTD9 | RGS10 | -0.506350041 | 7.51E-34 | 0.562102108 | 1.45E-05 | 1.84E-11 | MEblue |  |
| KCTD9 | RHOBTB2 | 0.526584403 | 6.06E-37 | -0.596274505 | 3.09E-06 | 3.50E-12 | MEblue |  |
| KCTD9 | WWC3 | 0.631948498 | 5.46E-57 | -0.665699135 | 7.25E-08 | 3.50E-12 | MEblue |  |
| KDELC2 | WWC3 | 0.610677639 | 2.51E-52 | -0.561355189 | 1.50E-05 | 3.50E-12 | MEblue |  |
| KDELC2 | ZNF185 | 0.523024076 | 2.20E-36 | -0.52175575 | 7.29E-05 | 6.89E-11 | MEblue |  |
| KPNA3 | WDFY2 | 0.5555672 | 9.36E-42 | -0.539495491 | 3.67E-05 | 3.50E-12 | MEblue |  |
| KRT15 | ST6GALNAC4 | 0.536590151 | 1.49E-38 | -0.622358865 | 8.40E-07 | 3.50E-12 | MEblue |  |
| KRT15 | TMEM139 | 0.535269623 | 2.45E-38 | -0.527786968 | 5.80E-05 | 2.06E-11 | MEblue |  |
| LAYN | WWC3 | 0.506084739 | 8.22E-34 | -0.535582692 | 4.29E-05 | 9.28E-11 | MEblue |  |
| LPCAT2 | MKX | 0.599189451 | 5.94E-50 | -0.520140222 | 7.74E-05 | 3.50E-12 | MEblue |  |
| LPCAT2 | NINL | 0.500057594 | 6.26E-33 | -0.545732527 | 2.86E-05 | 5.99E-11 | MEblue |  |
| LPCAT2 | NPTN | 0.687426586 | 4.75E-71 | -0.505568032 | 0.000131822 | 3.50E-12 | MEblue |  |
| LPCAT2 | NTN1 | 0.692915233 | 1.29E-72 | -0.510108922 | 0.000111968 | 3.50E-12 | MEblue |  |
| LPCAT2 | PPP3CB | 0.521645909 | 3.60E-36 | -0.555473383 | 1.92E-05 | 1.12E-11 | MEblue |  |
| LPCAT2 | PTGIS | 0.523149309 | 2.10E-36 | -0.514038498 | 9.70E-05 | 1.12E-10 | MEblue |  |
| LPCAT2 | SLC14A1 | 0.602645344 | 1.17E-50 | -0.577844265 | 7.27E-06 | 3.50E-12 | MEblue |  |
| LPCAT2 | SP8 | 0.521947489 | 3.23E-36 | -0.544098296 | 3.06E-05 | 2.06E-11 | MEblue |  |
| LPCAT2 | THSD4 | 0.650920729 | 1.82E-61 | -0.540959859 | 3.47E-05 | 3.50E-12 | MEblue |  |
| LPCAT2 | TMEM237 | 0.674237631 | 1.99E-67 | -0.540262251 | 3.56E-05 | 3.50E-12 | MEblue |  |
| LPCAT2 | VSNL1 | 0.516738505 | 2.06E-35 | -0.504525408 | 0.000136812 | 2.56E-10 | MEblue |  |
| MEIS2 | PAQR8 | 0.647753747 | 1.07E-60 | -0.526524919 | 6.09E-05 | 3.50E-12 | MEblue |  |
| MEIS2 | PDK4 | 0.506870988 | 6.29E-34 | -0.517765939 | 8.46E-05 | 2.20E-10 | MEblue |  |
| MEIS2 | TLR2 | 0.587025245 | 1.53E-47 | -0.515890382 | 9.07E-05 | 3.50E-12 | MEblue |  |
| MEIS2 | WWC3 | 0.595266338 | 3.66E-49 | -0.568276265 | 1.11E-05 | 3.50E-12 | MEblue |  |
| MEIS2 | ZFP92 | 0.584921299 | 3.91E-47 | -0.610990128 | 1.50E-06 | 3.50E-12 | MEblue |  |
| MKX | WWC3 | 0.521655493 | 3.59E-36 | -0.603352667 | 2.20E-06 | 3.50E-12 | MEblue |  |
| MKX | ZNF185 | 0.578616324 | 6.22E-46 | -0.51526748 | 9.28E-05 | 3.50E-12 | MEblue |  |
| MSMB | WFDC2 | 0.515336258 | 3.37E-35 | -0.64044855 | 3.17E-07 | 3.50E-12 | MEblue |  |
| NBL1 | QPRT | 0.518680569 | 1.04E-35 | -0.627956005 | 6.26E-07 | 3.50E-12 | MEblue |  |
| molecule.X | molecule.Y | r1 | p1 | r2 | p2 | FDR | Module |  |
| NINL | PAQR7 | 0.510245051 | 1.98E-34 | -0.503060562 | 0.000144115 | 4.21E-10 | MEblue |  |
| NPTN | WWC3 | 0.50093833 | 4.66E-33 | -0.612100822 | 1.42E-06 | 3.50E-12 | MEblue |  |
| NREP | UNC5B | 0.566680031 | 9.95E-44 | -0.510463126 | 0.00011054 | 1.12E-11 | MEblue |  |
| NTN1 | PAQR8 | 0.532427525 | 7.07E-38 | -0.596937177 | 3.00E-06 | 3.50E-12 | MEblue |  |
| NTN1 | QPRT | 0.558452306 | 2.92E-42 | -0.548244791 | 2.58E-05 | 3.50E-12 | MEblue |  |
| NTN1 | ST6GALNAC4 | 0.635137769 | 1.02E-57 | -0.560019841 | 1.58E-05 | 3.50E-12 | MEblue |  |
| PAQR7 | PPM1M | 0.501155295 | 4.34E-33 | -0.553299311 | 2.10E-05 | 4.40E-11 | MEblue |  |
| PAQR8 | SLC14A1 | 0.548209239 | 1.73E-40 | -0.62366366 | 7.85E-07 | 3.50E-12 | MEblue |  |
| PAQR8 | TMEM237 | 0.521020621 | 4.50E-36 | -0.62759299 | 6.38E-07 | 3.50E-12 | MEblue |  |
| PAQR8 | UST | 0.634098979 | 1.76E-57 | -0.575903948 | 7.93E-06 | 3.50E-12 | MEblue |  |
| PDCL3 | TSHZ3 | -0.502158531 | 3.10E-33 | 0.503389023 | 0.000142448 | 6.66E-10 | MEblue |  |
| PITX1 | PLLP | 0.509239074 | 2.80E-34 | -0.515297047 | 9.27E-05 | 2.20E-10 | MEblue |  |
| PITX1 | ST6GALNAC4 | 0.527291903 | 4.68E-37 | -0.553730734 | 2.06E-05 | 1.12E-11 | MEblue |  |
| PLA2G4A | THSD4 | 0.529630849 | 1.99E-37 | -0.58154788 | 6.15E-06 | 3.50E-12 | MEblue |  |
| PLLP | PPM1M | 0.539663331 | 4.67E-39 | -0.511971025 | 0.000104648 | 5.37E-11 | MEblue |  |
| PLLP | PRRX2 | 0.578723213 | 5.94E-46 | -0.549716397 | 2.43E-05 | 3.50E-12 | MEblue |  |
| PLLP | RARRES2 | 0.572350969 | 9.16E-45 | -0.532327311 | 4.87E-05 | 3.50E-12 | MEblue |  |
| PPM1M | WDR86 | 0.523554201 | 1.81E-36 | -0.542938534 | 3.20E-05 | 2.06E-11 | MEblue |  |
| PRNP | WWC3 | 0.525296715 | 9.67E-37 | -0.512949077 | 0.000100981 | 1.12E-10 | MEblue |  |
| PRRX2 | RHOBTB2 | 0.512588508 | 8.79E-35 | -0.614500947 | 1.26E-06 | 3.50E-12 | MEblue |  |
| PRRX2 | WDR86 | 0.545694595 | 4.60E-40 | -0.505046836 | 0.000134295 | 5.37E-11 | MEblue |  |
| PRRX2 | ZNF185 | 0.505717367 | 9.32E-34 | -0.589529838 | 4.26E-06 | 3.50E-12 | MEblue |  |
| PTGER2 | RHOBTB2 | 0.532042228 | 8.16E-38 | -0.529352027 | 5.46E-05 | 2.49E-11 | MEblue |  |
| PTGER2 | WWC3 | 0.524668924 | 1.21E-36 | -0.557761183 | 1.74E-05 | 1.12E-11 | MEblue |  |
| PTGER2 | ZNF185 | 0.572132877 | 1.00E-44 | -0.602340393 | 2.31E-06 | 3.50E-12 | MEblue |  |
| PTGIS | WWC3 | 0.500526566 | 5.35E-33 | -0.505751715 | 0.00013096 | 5.51E-10 | MEblue |  |
| PXDC1 | ST6GALNAC4 | 0.544538977 | 7.20E-40 | -0.571193189 | 9.77E-06 | 3.50E-12 | MEblue |  |
| QPRT | RPL39L | 0.502685319 | 2.60E-33 | -0.581632928 | 6.13E-06 | 8.40E-12 | MEblue |  |
| QPRT | SLC14A1 | 0.58784082 | 1.06E-47 | -0.536428175 | 4.15E-05 | 3.50E-12 | MEblue |  |
| QPRT | VSNL1 | 0.548011749 | 1.87E-40 | -0.513905974 | 9.75E-05 | 2.06E-11 | MEblue |  |
| RARB | WWC3 | 0.6192058 | 3.74E-54 | -0.565552524 | 1.25E-05 | 3.50E-12 | MEblue |  |
| RARB | ZFP92 | 0.606751903 | 1.67E-51 | -0.532669939 | 4.80E-05 | 3.50E-12 | MEblue |  |
| RHOBTB2 | SORBS3 | 0.614965742 | 3.08E-53 | -0.661763149 | 9.21E-08 | 3.50E-12 | MEblue |  |
| SEPT7 | WWC3 | 0.581497895 | 1.77E-46 | -0.517625388 | 8.50E-05 | 3.50E-12 | MEblue |  |
| SLC14A1 | ST6GALNAC4 | 0.564645996 | 2.32E-43 | -0.584672632 | 5.33E-06 | 3.50E-12 | MEblue |  |
| SLC14A1 | ZNF185 | 0.669184505 | 4.33E-66 | -0.575177683 | 8.19E-06 | 3.50E-12 | MEblue |  |
| SLC16A2 | WWC3 | 0.620661885 | 1.80E-54 | -0.518953666 | 8.09E-05 | 3.50E-12 | MEblue |  |
| SLC16A2 | ZFP92 | 0.605477045 | 3.07E-51 | -0.514556875 | 9.52E-05 | 3.50E-12 | MEblue |  |
| ST6GALNAC4 | TRPV4 | 0.61553765 | 2.32E-53 | -0.515813258 | 9.09E-05 | 3.50E-12 | MEblue |  |
| ST6GALNAC4 | VSNL1 | 0.600650119 | 3.00E-50 | -0.500447308 | 0.000158033 | 3.50E-12 | MEblue |  |
| THSD4 | WWC3 | 0.6100736 | 3.37E-52 | -0.547725042 | 2.64E-05 | 3.50E-12 | MEblue |  |
| molecule.X | molecule.Y | r1 | p1 | r2 | p2 | FDR | Module |  |
| TMEM237 | WWC3 | 0.527303862 | 4.66E-37 | -0.611409071 | 1.47E-06 | 3.50E-12 | MEblue |  |
| TMEM237 | ZNF185 | 0.54335057 | 1.14E-39 | -0.596819221 | 3.02E-06 | 3.50E-12 | MEblue |  |
| TMEM43 | WWC3 | 0.67058994 | 1.85E-66 | -0.566521894 | 1.20E-05 | 3.50E-12 | MEblue |  |
| TSHZ3 | WWC3 | 0.573334832 | 6.03E-45 | -0.507849751 | 0.000121475 | 1.12E-11 | MEblue |  |
| TTC39B | WWC3 | 0.520294571 | 5.84E-36 | -0.501696137 | 0.000151236 | 2.56E-10 | MEblue |  |
| UST | ZNF185 | 0.541367698 | 2.44E-39 | -0.569019685 | 1.07E-05 | 3.50E-12 | MEblue |  |
| WWC3 | WWTR1 | 0.527287934 | 4.69E-37 | -0.505072488 | 0.000134173 | 1.46E-10 | MEblue |  |
| ACOT7 | PDRG1 | 0.552134715 | 3.68E-41 | -0.651821691 | 1.66E-07 | 2.11E-11 | MEmediumpurple3 |  |
| ANAPC15 | ZNF787 | 0.55196517 | 3.93E-41 | -0.525336978 | 6.37E-05 | 4.82E-11 | MEmediumpurple3 |  |
| C4orf48 | RAB11FIP2 | -0.609550142 | 4.34E-52 | 0.500576933 | 0.000157315 | 2.11E-11 | MEmediumpurple3 |  |
| C4orf48 | ZNF784 | 0.500977547 | 4.60E-33 | -0.540144033 | 3.58E-05 | 3.51E-10 | MEmediumpurple3 |  |
| CDKN2D | PDRG1 | 0.513994199 | 5.39E-35 | -0.50705549 | 0.00012499 | 9.69E-10 | MEmediumpurple3 |  |
| CPNE7 | MAPK12 | 0.520713683 | 5.03E-36 | -0.513208255 | 0.000100029 | 3.51E-10 | MEmediumpurple3 |  |
| FAM69B | LCN12 | 0.557321748 | 4.62E-42 | -0.578307564 | 7.12E-06 | 2.11E-11 | MEmediumpurple3 |  |
| FAM98C | LCN12 | 0.539975517 | 4.14E-39 | -0.50225323 | 0.000148291 | 2.53E-10 | MEmediumpurple3 |  |
| FYCO1 | ZNF771 | -0.517222169 | 1.74E-35 | 0.609491851 | 1.62E-06 | 2.11E-11 | MEmediumpurple3 |  |
| HES4 | LCN12 | 0.531747091 | 9.10E-38 | -0.505197086 | 0.000133578 | 3.51E-10 | MEmediumpurple3 |  |
| HES4 | PDRG1 | 0.516897506 | 1.95E-35 | -0.502912734 | 0.000144872 | 1.07E-09 | MEmediumpurple3 |  |
| HM13 | MON1A | 0.578114012 | 7.73E-46 | -0.570302021 | 1.02E-05 | 2.11E-11 | MEmediumpurple3 |  |
| LCN12 | PDDC1 | 0.527971552 | 3.65E-37 | -0.519306485 | 7.99E-05 | 2.17E-10 | MEmediumpurple3 |  |
| LCN12 | RNF215 | 0.536393326 | 1.61E-38 | -0.522648139 | 7.05E-05 | 1.64E-10 | MEmediumpurple3 |  |
| LCN12 | TNK2 | 0.58852102 | 7.84E-48 | -0.509091713 | 0.000116161 | 2.11E-11 | MEmediumpurple3 |  |
| LRSAM1 | PDRG1 | 0.501822806 | 3.47E-33 | -0.513229758 | 1.00E-04 | 1.11E-09 | MEmediumpurple3 |  |
| MON1A | WDR45 | 0.542959042 | 1.32E-39 | -0.539789909 | 3.63E-05 | 4.82E-11 | MEmediumpurple3 |  |
| NOP56 | SLC6A8 | 0.510231377 | 1.99E-34 | -0.606130347 | 1.92E-06 | 2.11E-11 | MEmediumpurple3 |  |
| PCBP4 | PDRG1 | 0.583430838 | 7.56E-47 | -0.557727853 | 1.74E-05 | 2.11E-11 | MEmediumpurple3 |  |
| PDRG1 | SH3GL1 | 0.528336951 | 3.19E-37 | -0.520762961 | 7.56E-05 | 2.17E-10 | MEmediumpurple3 |  |
| PDRG1 | TNIP2 | 0.61007224 | 3.37E-52 | -0.522351589 | 7.13E-05 | 2.11E-11 | MEmediumpurple3 |  |
| SLC25A10 | ZNF771 | 0.530980054 | 1.21E-37 | -0.548337118 | 2.57E-05 | 4.82E-11 | MEmediumpurple3 |  |
| TOR1AIP1 | ZNF771 | -0.529426812 | 2.14E-37 | 0.503851639 | 0.000140128 | 3.51E-10 | MEmediumpurple3 |  |
